# Development and validation of an interpretable machine learning model for predicting multidrug-resistant infections in ICU patients

**DOI:** 10.3389/fcimb.2026.1807363

**Published:** 2026-08-10

**Authors:** Yimeng Yang, Jianrui Lv, Junbin Tian, Shuo Yu, Guangdong Wang, Lei Ma

**Affiliations:** 1 Anesthesia and Surgical Center, The Second Affiliated Hospital of Xi’an Jiaotong University, Xi’an, Shaanxi, China; 2 Department of General Surgery, The Second Affiliated Hospital of Xi’an Jiaotong University, Xi’an, Shaanxi, China; 3 Department of Respiratory and Critical Medicine, The First Affiliated Hospital of Xi’an Jiaotong University, Xi’an, Shaanxi, China

**Keywords:** intensive care units, machine learning, multidrug-resistant infections, prediction model, random forest

## Abstract

**Background:**

Multidrug-resistant (MDR) infections in intensive care units (ICUs) are difficult to recognize early. This study developed and validated an interpretable machine-learning model for early MDR risk prediction in ICU patients.

**Methods:**

A retrospective cohort was built from the Medical Information Mart for Intensive Care IV (MIMIC-IV, n=48,501) for model development with internal validation (7:3 split) and from MIMIC-III (n=38,583) for temporal validation. Candidate predictors at ICU admission included demographics, vital signs, laboratory indices, comorbidities, therapeutic interventions, and severity scores. Missing values were imputed using missForest. Highly correlated predictors were removed using correlation screening. Feature selection used the intersection of Least Absolute Shrinkage and Selection Operator regression and Boruta. Data imbalance was handled by Random Undersampling. Eight models were constructed including Logistic Regression, K-Nearest Neighbors, Decision Tree, Random Forest (RF), Extreme Gradient Boosting, Light Gradient Boosting Machine, Support Vector Machine, and Neural Network. Hyperparameters were optimized using grid search and cross-validation. Model performance, calibration, and clinical utility were evaluated. Model interpretability used SHapley Additive exPlanations (SHAP). A web calculator was implemented using Shiny in R.

**Results:**

RF achieved the best overall performance in internal validation (AUC 0.784, 95% CI 0.769-0.798) and remained stable in temporal validation (AUC 0.778, 95% CI 0.771-0.786). The final model included 19 predictors. SHAP identified enteral nutrition, Acute Physiology Score III, stage of acute kidney injury, blood urea nitrogen, opioids use, hemoglobin, and platelet as the leading contributors. A web-based calculator was created to output individualized risk with SHAP-based explanations.

**Conclusions:**

We developed and validated an interpretable RF model for early prediction of MDR infections in ICU patients and translated it into a web-based calculator with SHAP explanations, enabling rapid bedside risk stratification to support timely antimicrobial stewardship and resource allocation.

## 1 Introduction

Multidrug-resistant (MDR) bacterial infections, defined as acquired non-susceptibility to at least one agent in three or more antimicrobial categories (Magiorakos et al., 2012), have emerged as a defining crisis of twenty-first century medicine. Global Research on Antimicrobial Resistance (GARM) program reported that methicillin-resistant Staphylococcus aureus (MRSA), the most widely recognized MDR pathogen, was directly responsible for approximately 130,000 deaths globally in 2021, representing a more than two-fold increase from the 57,200 MRSA-attributable deaths recorded in 1990. While carbapenem-resistant Gram-negative bacteria, which are MDR or extensively drug-resistant (XDR) by definition, caused an estimated 216,000 direct deaths in 2021, rising from 127,000 three decades earlier, and have since been designated the highest-priority resistance threat by the World Health Organization (WHO) (GBD 2021 Antimicrobial Resistance Collaborators, 2024). These MDR pathogens collectively constitute a substantial and disproportionate component of the 1.27 million deaths directly attributable to bacterial resistance worldwide in 2019 and the 4.95 million deaths in which resistance was a contributing factor (Antimicrobial Resistance Collaborators, 2022), and it is forecasted that annual resistance-attributable mortality would reach 1.91 million direct deaths by 2050 and accumulate to over 39 million deaths between 2025 and 2050 (GBD 2021 Antimicrobial Resistance Collaborators, 2024). The macroeconomic consequences of this trajectory are equally sobering. The World Bank has estimated that uncontrolled drug resistance could impose annual global GDP losses of $1.0-3.4 trillion by 2050, with the burden falling most severely on low- and middle-income countries, where inadequate laboratory infrastructure, unrestricted antibiotic access, and fragile infection prevention capacity accelerate the selection and dissemination of resistant strains (Jonas et al., 2017). Within this global landscape, the intensive care unit (ICU) has emerged as the clinical setting where MDR infection risk is most concentrated, clinical consequences most severe, and the need for earlier recognition most urgent. The convergence of critically ill immunocompromised hosts, high-frequency invasive procedures, and intense antibiotic selective pressure creates an environment uniquely permissive for the emergence, amplification, and nosocomial transmission of MDR pathogens. Epidemiological data indicate a staggering burden, with MDR prevalence in ICUs ranging from 15.1% to 51.9% worldwide (Magira et al., 2018; Aliyu et al., 2022; Ibrahim et al., 2024; Cornistein et al., 2025). The clinical burden observed in ICU settings is predominantly driven by a core group of organisms collectively designated the ESKAPE pathogens, including *Enterococcus faecium*, *Staphylococcus aureus*, *Klebsiella pneumoniae*, *Acinetobacter baumannii*, *Pseudomonas aeruginosa*, and *Enterobacter* spp., alongside carbapenem-resistant Enterobacteriaceae (CRE) and extended-spectrum β-lactamase (ESBL)-producing strains (De Oliveira et al., 2020). The 2024 WHO Bacterial Priority Pathogens List designates carbapenem-resistant *A. baumannii*, carbapenem-resistant *P. aeruginosa*, and third-generation cephalosporin-resistant *K. pneumoniae* in the highest-priority “Critical” tier, reflecting both the life-threatening severity of the infections these organisms cause and the extreme therapeutic challenges posed by dramatically narrowed antibiotic options (World Health Organization, 2024).These infections are consistently associated with profound clinical and economic consequences. Compared to susceptible infections, MDR cases lead to significantly higher mortality rates, reported as 49.1% vs. 29.6% in septic patients, with some outbreaks showing a hazard ratio for death as high as 7.11 (95% CI, 1.52-33.2) (Munier et al., 2019; Aliyu et al., 2022). Furthermore, MDR infections significantly prolong hospitalization, extending the length of stay by 6.7 to 14 days (Tabak et al., 2019; Cornistein et al., 2025). The financial strain is equally severe, with studies reporting incremental costs exceeding $ 20000 per case and significantly higher 30-day readmission rates (Tabak et al., 2019). Consequently, rapid identification of high-risk patients is imperative to improve survival and optimize resource allocation.

The gold standard for diagnosing MDR infections relies on microbiological cultures and antimicrobial susceptibility testing. However, these methods are inherently time-consuming, typically requiring 48 to 72 hours to yield definitive results. This diagnostic lag creates a critical “blind window” where clinicians must rely on empirical antibiotic therapy, inappropriate use during this period risks treatment failure, while excessive broad-spectrum use further drives resistance selection. Therefore, constructing early prediction tools is of great clinical significance, not only to guide precise antimicrobial stewardship but also to improve patient prognosis and optimize the allocation of healthcare resources. Recent years have brought meaningful advances across the spectrum of MDR infection management. Matrix-assisted laser desorption/ionization time-of-flight mass spectrometry has substantially reduced pathogen identification time from the traditional hours to under one hour following blood culture positivity, and has been associated with reductions in time to effective therapy and in-hospital mortality in bacteremic patients (Huang et al., 2013). A study reported that deployment of rapid multiplex PCR, capable of simultaneously detecting common pathogens and key resistance determinants within 1–2 hours, combined with real-time stewardship guidance significantly improved antibiotic management decisions and reduced time to optimal treatment in hospitalized patients with positive blood cultures (Banerjee et al., 2015). Despite these advances, both technologies remain fundamentally contingent on the availability of a prior positive culture specimen, precluding their application for pre-culture risk stratification at the time of ICU admission. The regulatory approval of novel β-lactam/β-lactamase inhibitor combinations has materially expanded the therapeutic armamentarium against CRE and XDR organisms (Bassetti et al., 2021; Tamma et al., 2023). Ceftazidime-avibactam achieves significantly lower 30-day mortality compared with colistin-based regimens for CRE infections (van Duin et al., 2018), while structured antibiotic stewardship programs (ASPs) have demonstrated consistent reductions in inappropriate antibiotic use, resistance selection pressure, and infection-related harm in ICU settings when anchored to timely microbiological intelligence (Barlam et al., 2016). However, all the new methods remain contingent upon resistance phenotyping data that are unavailable during the critical 48- to 72-hour diagnostic window. This persistent diagnostic-therapeutic paradox underscores the need of pre-culture risk stratification tools capable of guiding individualized empirical antimicrobial decisions from the moment of ICU admission. Although machine learning (ML) has emerged to bridge this gap, a recent systematic review highlighted that current models are often insufficient for clinical application due to high risks of bias and lack of external validation (Pu et al., 2025). Specifically, existing models often exhibit poor generalizability, with performance declining significantly when tested on independent cohorts (Li et al., 2024), or are limited to specific subpopulations such as neuro-critical care (Jiang et al., 2023; Yang et al., 2025) or ventilator-associated pneumonia (Cui et al., 2025). Furthermore, few studies have translated complex algorithms into interpretable, deployable bedside tools, leaving most models as theoretical “black boxes”. Consequently, there is an urgent unmet need for a robust, generalizable, and user-friendly prediction tool to guide early precise antimicrobial stewardship.

This study aimed to develop and validate a robust, interpretable ML-based model for the early prediction of MDR infections in ICU patients. We leveraged the extensive Medical Information Mart for Intensive Care (MIMIC)-IV database for model development and internal validation and performed strict temporal validation using MIMIC-III database to ensure generalizability. Methodologically, we identified Random Forest as the optimal algorithm from eight ML-based models and integrated SHapley Additive exPlanations (SHAP) to dismantle the “black-box” nature of the model, providing transparent, visual interpretations of individual risk predictions. Furthermore, to facilitate translation into clinical practice, we developed a user-friendly web-based risk calculator. This comprehensive framework is designed to empower clinicians with a precise, easy-to-use tool for early risk stratification, ultimately guiding timely and appropriate antimicrobial stewardship.

## 2 Materials and methods

### 2.1 Data source

Data for this retrospective analysis were obtained from the MIMIC-III (version 1.4) and MIMIC-IV (version 3.1) databases. Access to these databases was granted following the completion of the required Collaborative Institutional Training Initiative Program course (Guangdong Wang, Record ID: 60106105). The establishment of both databases was approved by the Institutional Review Boards at the Massachusetts Institute of Technology and Beth Israel Deaconess Medical Center. Given the retrospective design and the utilization of de-identified records, the requirement for individual informed consent was waived. All study procedures were conducted in strict accordance with the ethical principles outlined in the Declaration of Helsinki.

### 2.2 Patient selection

The study cohort was curated using Structured Query Language (SQL) scripts applied to the MIMIC-III and MIMIC-IV databases. Patients were enrolled based on the following eligibility criteria: (1) initial admission to the ICU; (2) ICU length of stay≥24 hours; and (3) age ranging from 18 to 89 years. The outcome variable was the identification of multidrug-resistant (MDR) infections. MDR was defined as acquired non-susceptibility to at least one agent in three or more antimicrobial categories based on microbiology culture and antimicrobial susceptibility testing results obtained during the ICU stay (Magiorakos et al., 2012). Patients whose MDR results were obtained within 24 hours of ICU admission or before ICU were excluded.

### 2.3 Variables extraction

Baseline demographic characteristics, clinical parameters, and laboratory data within the first 24 hours after ICU admission were extracted using SQL scripts. To ensure temporal consistency and reduce the risk of data leakage, all predictor variables were extracted within the first 24 hours after ICU admission. The extracted variables were categorized as follows: (1) Demographics: age, gender, race, and marital status. (2) Vital signs and physiological parameters: heart rate, systolic and diastolic blood pressure (SBP, DBP), respiratory rate, temperature, height, weight, and urine output. (3) Laboratory indices: complete blood count including white blood cell (WBC) count, red blood cell (RBC) count, hemoglobin, hematocrit, and platelets; inflammatory markers such as C-reactive protein (CRP); liver function tests including total bilirubin, aspartate aminotransferase (AST), alkaline phosphatase (ALP), alanine aminotransferase (ALT), gamma-glutamyl transferase (GGT), total protein, globulin, and albumin; kidney function markers including blood urea nitrogen (BUN) and creatinine. Other metabolic and coagulation profiles included lactate dehydrogenase (LDH), glucose, electrolytes (sodium, potassium, calcium, chloride), anion gap, fibrinogen, prothrombin time (PT), partial thromboplastin time (PTT), international normalized ratio (INR), and D-dimer. Arterial blood gas analysis parameters included partial pressure of carbon dioxide (PCO2) and oxygen (PO2), oxygen saturation (SO2), base excess, bicarbonate, pH, and lactate. (4) Comorbidities: liver disease, renal disease, malignant cancer, diabetes, chronic pulmonary disease, congestive heart failure, and acute kidney injury (AKI) and AKI stage. (5) Therapeutic interventions: pharmacological treatments included neuromuscular blocking agents (NMBAs), sedatives, opioids, non-steroidal anti-inflammatory drugs (NSAIDs), statins, systemic glucocorticoids, inhaled corticosteroids (ICS), vasoactive drugs, immunosuppressors, and biological agents. Clinical procedures and support encompassed continuous renal replacement therapy (CRRT), mechanical ventilation, enteral and parenteral nutrition, as well as the utilization of invasive lines, and peripheral lines. (6) Severity of illness scores: Systemic Inflammatory Response Syndrome (SIRS) criteria, Oxford Acute Severity of Illness Score (OASIS), Logistic Organ Dysfunction System (LODS), Acute Physiology Score III (APSIII), Glasgow Coma Scale (GCS), Simplified Acute Physiology Score II (SAPSII), and the Sequential Organ Failure Assessment (SOFA) score. (7) Clinical outcomes: The extracted outcome measures included length of stay in the hospital and ICU, hospital and ICU mortality, as well as 30-day, 90-day, and 180-day survival status and follow-up times post-ICU admission.

### 2.4 Statistical analysis

Continuous variables were expressed as mean ± standard deviation (SD) or median with interquartile range (IQR) based on their distribution normality, while categorical variables were presented as frequencies and percentages. Comparisons between the MDR and non-MDR groups were performed using the Student’s t-test or Mann-Whitney U test for continuous variables, and the Chi-square test or Fisher’s exact test for categorical variables.

**Figure 1:**
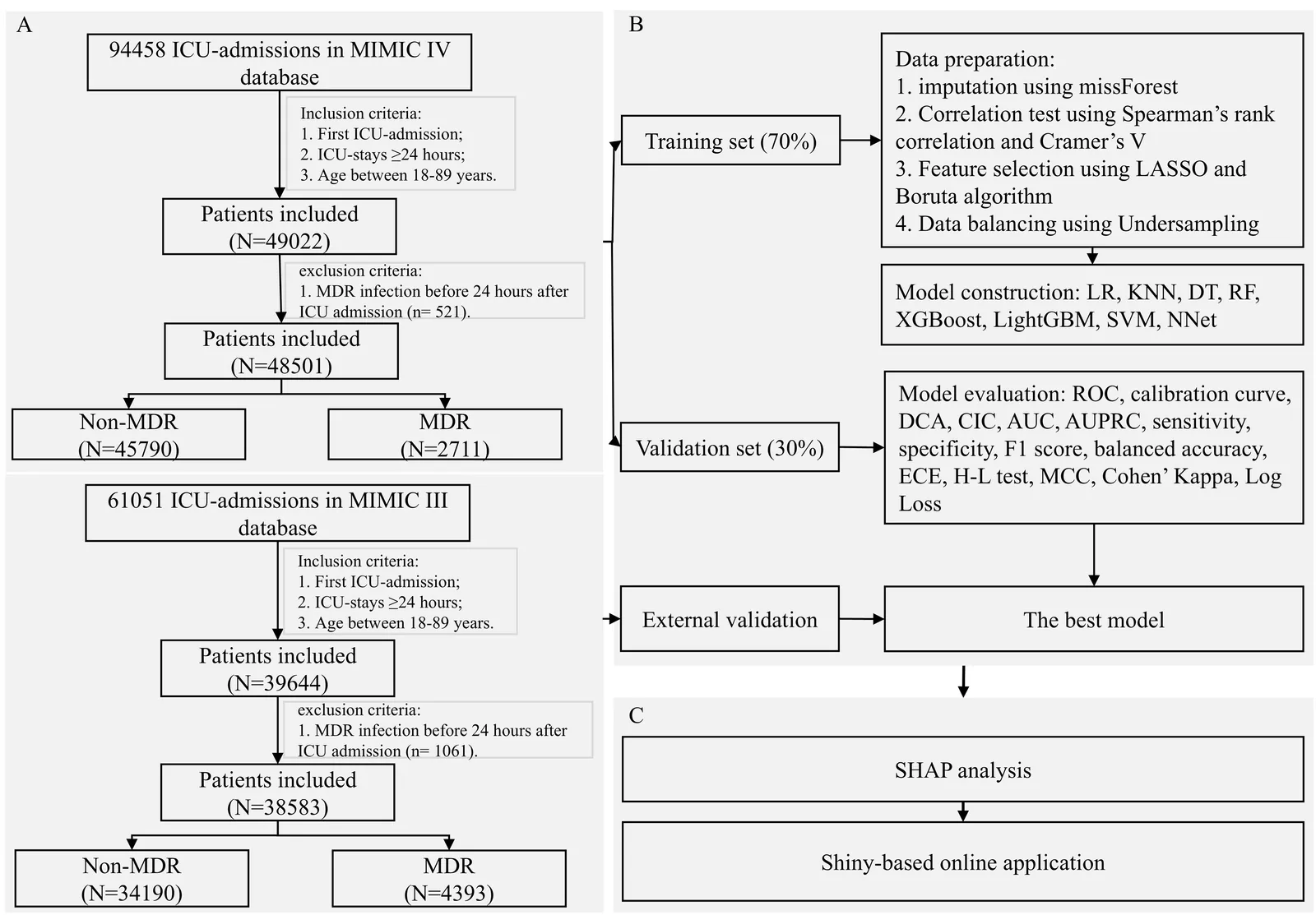
Study flowchart and model-development pipeline for early prediction of multidrug-resistant (MDR) infections in ICU patients. **(A)** Patient selection flowchart for the MIMIC-IV development cohort and MIMIC-III temporal validation cohort. **(B)** Model development pipeline, including data preparation, model construction, and model evaluation. **(C)** Post-modeling procedures, including SHAP-based interpretability analysis and deployment of the Shiny-based online application. AUC, area under the receiver operating characteristic curve; AUPRC, area under the precision–recall curve; CIC, clinical impact curve; DCA, decision curve analysis; DT, Decision Tree; ECE, Expected Calibration Error; H-L test, Hosmer-Lemeshow Test; ICU, intensive care unit; KNN, K-Nearest Neighbors; LASSO, Least Absolute Shrinkage and Selection Operator; LightGBM, Light Gradient Boosting Machine; LR, Logistic Regression; MCC, Matthews Correlation Coefficient; MDR, multidrug-resistant; MIMIC, Medical Information Mart for Intensive Care; NNet, Neural Network; RF, Random Forest; ROC, receiver operating characteristic; SHAP, SHapley Additive exPlanations; SVM, Support Vector Machine; XGBoost, Extreme Gradient Boosting.

The study was conducted strictly according to the flowchart in **Figure 1**. Variables with a missing rate exceeding 25% were excluded from the analysis (**Supplementary Tables 1**, **2**). The MIMIC-IV cohort was then randomly divided into a training set and an internal validation set at a ratio of 7:3. All subsequent preprocessing steps for model development were performed based on the training set. For variables with less than 25% missing data, the missForest algorithm was employed to impute missing values. MissForest is a non-parametric, random forest-based imputation algorithm that handles both continuous and categorical variables simultaneously, makes no distributional assumptions, and has been demonstrated to outperform other imputation methods (including multiple imputation by chained equations, MICE) in various benchmarking studies, particularly in complex clinical datasets with mixed variable types (Stekhoven and Bühlmann, 2012). To evaluate the stability of the imputation, missForest was independently executed five times with 5 distinct random seeds. The coefficient of variation (CV) of the imputed means was calculated for each continuous variable to quantify cross-seed variability (**Supplementary Table 3**). Additionally, MICE (m = 5) was also performed, and the optimal model was re-evaluated on the MICE-imputed data (**Supplementary Figure 1**). To mitigate multicollinearity, Spearman’s rank correlation analysis was conducted in the training set for continuous variables, and Cramér’s V was calculated for categorical variables (**Supplementary Figures 2**, **3**). Variable pairs with a Spearman correlation coefficient > 0.6 or Cramér’s V > 0.5 were identified, and the variable showing a weaker correlation with the outcome (MDR infections) was removed.

Within the training set, feature selection was then performed using the intersection of variables identified by Least Absolute Shrinkage and Selection Operator (LASSO) regression and the Boruta algorithm. After the final predictor set had been determined, random undersampling was applied only in the training set to the selected predictors and outcome, using a 1:1 ratio between MDR and non-MDR cases. This technique involves randomly removing samples from the majority class to match the size of the minority class, thereby preventing the model from being biased toward the majority class while maintaining computational efficiency. The resulting balanced training dataset was then used for model construction. The internal validation set was not involved in feature selection or undersampling. Subsequently, eight ML-based models were constructed using the balanced training data, including: Logistic Regression (LR), K-Nearest Neighbors (KNN), Decision Tree (DT), Random Forest (RF), Extreme Gradient Boosting (XGBoost), Light Gradient Boosting Machine (LightGBM), Support Vector Machine (SVM), and Neural Network (NNet). Hyperparameters were optimized using grid search strategies coupled with 10-fold cross-validation or an inner validation subset split from the training set to maximize the area under the receiver operating characteristic (ROC) curve, displayed in **Supplementary Table 4**. The internal validation set was utilized for model evaluation. The discriminative performance was comprehensively assessed using the ROC curve, area under the precision-recall curve (AUPRC), sensitivity, specificity, F1-score, and balanced accuracy. Additionally, Matthews Correlation Coefficient (MCC) and Cohen’s Kappa were computed to evaluate overall classification quality. Probabilistic accuracy was assessed using Log Loss and the Brier score. Calibration was evaluated using calibration curves, the Expected Calibration Error (ECE), and the Hosmer–Lemeshow goodness-of-fit test. Additionally, decision curve analysis (DCA), and clinical impact curves (CIC) were used to evaluate model clinical utility. Because the internal validation set retained the original, imbalanced MDR prevalence, a prior probability shift (log-odds offset) correction was applied to the raw predicted probabilities to recalibrate them to the true population-level prevalence before calculating probability-dependent metrics (Log Loss, Brier score, ECE, the Hosmer–Lemeshow test, calibration curves, DCA, and CIC), whereas rank-based metrics (AUC and AUPRC) were unaffected by this transformation and were calculated from the original predicted probabilities. Based on these performance metrics, the optimal model was selected. Finally, the MIMIC-III dataset was employed to strictly perform temporal validation on the best-performing model to test its generalizability. Furthermore, the CareVue clinical information system, a subset of MIMIC-III, mainly includes ICU admissions before 2008, which allowed us to examine model performance across different temporal periods and partially evaluate robustness to temporal shifts in patient characteristics and clinical practice. SHAP values were calculated to quantify the contribution of each feature to the prediction. Finally, a user-friendly web-based calculator was developed using the Shiny package in R to facilitate clinical application.

All statistical analyses were performed using R software (version 4.4.2). A two-sided p-value < 0.05 was considered statistically significant.

## 3 Results

### 3.1 Baseline characteristics of ICU patients stratified by MDR status

A total of 48501 eligible patients in the full MIMIC-IV cohort were included in the final analysis. Among them, 2711 (5.6%) patients were identified with MDR infections. The baseline characteristics of the study population, stratified by MDR status, are summarized in **Table 1**.

**Table 1:** Baseline characteristics of ICU patients in MIMIC-IV according to multidrug-resistant (MDR) infections status.

Variables	Non-MDR (n=45790)	MDR (n=2711)	p-value
Age, years	63.75 (15.90)	64.35 (15.35)	0.055
Gender, male, n (%)	26748 (58.4)	1448 (53.4)	<0.001
Race, n (%)			0.01
White	29935 (65.4)	1691 (62.4)	
Black	4096 (8.9)	262 (9.7)	
Yellow	1384 (3.0)	80 (3.0)	
Others	10375 (22.7)	678 (25.0)	
Marital status, n (%)			<0.001
Single	12163 (26.6)	825 (30.4)	
Married	21432 (46.8)	1036 (38.2)	
Divorced	3227 (7.0)	252 (9.3)	
Widowed	4177 (9.1)	277 (10.2)	
Unknown	4791 (10.5)	321 (11.8)	
Temperature, °C	36.68 (0.78)	36.79 (0.95)	<0.001
Respiratory rate, bpm	18.82 (5.85)	20.37 (6.29)	<0.001
MBP, mmHg	85.00 (18.26)	83.02 (19.42)	<0.001
DBP, mmHg	69.24 (17.59)	68.21 (18.57)	0.003
SBP, mmHg	124.58 (24.08)	122.50 (25.81)	<0.001
Heart rate, bpm	87.38 (19.71)	93.53 (21.79)	<0.001
Urine output, ml	1909.51 (1240.09)	1632.58 (1210.79)	<0.001
WBC, K/uL	12.21 (8.98)	13.97 (14.53)	<0.001
RBC, m/uL	3.63 (0.78)	3.48 (0.79)	<0.001
Hemoglobin, mg/dl	10.86 (2.27)	10.35 (2.23)	<0.001
Hematocrit, %	32.85 (6.67)	31.67 (6.69)	<0.001
Platelet, K/uL	202.98 (100.87)	215.22 (125.85)	<0.001
BUN, mg/dl	23.27 (20.01)	31.75 (26.28)	<0.001
Creatinine, mg/dl	1.30 (1.49)	1.65 (1.64)	<0.001
Glucose, mg/dl	142.70 (73.41)	153.52 (84.78)	<0.001
Sodium, mEq/L	138.29 (4.96)	138.11 (6.33)	0.063
Potassium, mEq/L	4.19 (0.70)	4.24 (0.80)	<0.001
Calcium, mEq/L	8.36 (0.80)	8.23 (0.99)	<0.001
Anion gap, mEq/L	13.92 (4.27)	14.83 (4.49)	<0.001
Chloride, mEq/L	104.48 (6.29)	103.59 (7.44)	<0.001
PT, s	15.61 (7.33)	17.22 (9.58)	<0.001
PTT, s	36.77 (21.27)	38.90 (22.51)	<0.001
INR	1.43 (0.70)	1.57 (0.85)	<0.001
Liver disease, n (%)	4994 (10.9)	568 (21.0)	<0.001
Renal disease, n (%)	7766 (17.0)	647 (23.9)	<0.001
Malignant cancer, n (%)	5866 (12.8)	434 (16.0)	<0.001
Diabetes, n (%)	13071 (28.5)	943 (34.8)	<0.001
Chronic pulmonary disease, n (%)	10373 (22.7)	804 (29.7)	<0.001
Congestive heart failure, n (%)	10677 (23.3)	805 (29.7)	<0.001
AKI, n (%)	32349 (70.6)	2298 (84.8)	<0.001
AKI stage, n (%)			<0.001
1	9074 (19.8)	348 (12.8)	
2	16220 (35.4)	954 (35.2)	
3	7055 (15.4)	996 (36.7)	
SIRS	2.55 (0.94)	2.84 (0.91)	<0.001
OASIS	30.88 (8.43)	35.16 (8.78)	<0.001
LODS	4.14 (2.82)	5.92 (3.22)	<0.001
APSIII	41.60 (19.84)	54.55 (22.81)	<0.001
GCS	14.88 (0.61)	14.84 (0.78)	<0.001
SAPSII	34.75 (13.72)	41.61 (14.76)	<0.001
SOFA	4.27 (3.16)	5.94 (3.81)	<0.001
NMBA, n (%)	2750 (6.0)	533 (19.7)	<0.001
Sedative, n (%)	28548 (62.3)	2017 (74.4)	<0.001
Opioids, n (%)	20628 (45.0)	1860 (68.6)	<0.001
NSAIDs, n (%)	32920 (71.9)	2060 (76.0)	<0.001
Statins, n (%)	16399 (35.8)	678 (25.0)	<0.001
Systemic glucocorticoids, n (%)	8842 (19.3)	818 (30.2)	<0.001
ICS, n (%)	3664 (8.0)	272 (10.0)	<0.001
Vasoactive, n (%)	15321 (33.5)	1311 (48.4)	<0.001
Immunosuppressor, n (%)	1027 (2.2)	107 (3.9)	<0.001
Biological agent, n (%)	38784 (84.7)	2505 (92.4)	<0.001
CRRT, n (%)	1516 (3.3)	357 (13.2)	<0.001
Ventilation, n (%)	35391 (77.3)	2379 (87.8)	<0.001
Enteral nutrition, n (%)	6406 (14.0)	1140 (42.1)	<0.001
Parenteral nutrition, n (%)	516 (1.1)	166 (6.1)	<0.001
Invasive Lines, n (%)	27368 (59.8)	2053 (75.7)	<0.001
Peripheral Lines, n (%)	39694 (86.7)	2259 (83.3)	<0.001
Los hospital, days	10.10 (9.92)	26.57 (24.31)	<0.001
Hospital mortality, n (%)	4306 (9.4)	560 (20.7)	<0.001
Los ICU, days	3.94 (4.84)	10.33 (12.37)	<0.001
ICU mortality, n (%)	3080 (6.7)	326 (12.0)	<0.001
30-day survival time, days	27.46 (7.36)	26.63 (7.63)	<0.001
30-day mortality, n (%)	5527 (12.1)	541 (20.0)	<0.001
90-day survival time, days	78.85 (27.11)	70.31 (31.68)	<0.001
90-day mortality, n (%)	7333 (16.0)	849 (31.3)	<0.001
180-day survival time, days	153.16 (59.36)	129.15 (71.24)	<0.001
180-day mortality, n (%)	8546 (18.7)	1011 (37.3)	<0.001

AKI, acute kidney injury; APSIII, Acute Physiology Score III; BUN, blood urea nitrogen; DBP, diastolic blood pressure; GCS, Glasgow Coma Scale; ICS, inhaled corticosteroids; INR, international normalized ratio; ICU, intensive care unit; LODS, Logistic Organ Dysfunction System; LOS, length of stay; MBP, mean blood pressure; MDR, multidrug-resistant; NMBA, neuromuscular blocking agent; NSAIDs, non-steroidal anti-inflammatory drugs; OASIS, Oxford Acute Severity of Illness Score; PT, prothrombin time; PTT, partial thromboplastin time; SAPSII, Simplified Acute Physiology Score II; SBP, systolic blood pressure; SIRS, Systemic Inflammatory Response Syndrome; SOFA, Sequential Organ Failure Assessment; WBC, white blood cell.

Compared to the non-MDR group, patients in the MDR group had a lower proportion of males (53.4% vs. 58.4%, p < 0.001). Clinically, the MDR group presented with a significantly higher severity of illness upon ICU admission. This was evidenced by higher scores across all severity indices, including SIRS, OASIS, LODS, APSIII, SAPSII, SOFA (all p < 0.001). Physiologically, MDR patients exhibited greater instability, characterized by higher heart rates and respiratory rates, but lower blood pressure compared to non-MDR patients. Laboratory analysis revealed that the MDR group had worse renal function (higher BUN and creatinine), elevated inflammatory markers (higher WBC), and prolonged coagulation times (PT, PTT, INR). Regarding comorbidities, the burden of chronic disease was significantly heavier in the MDR group. Diabetes (34.8% vs. 28.5%), congestive heart failure (29.7% vs. 23.3%), chronic pulmonary disease (29.7% vs. 22.7%), and renal disease (23.9% vs. 17.0%) were all more prevalent in MDR patients (p < 0.001). Additionally, the incidence of AKI was striking in the MDR group (84.8% vs. 70.6%), with a significantly higher proportion of patients progressing to AKI Stage 3 (36.7% vs. 15.4%). Consequently, patients with MDR infections required more intensive therapeutic interventions, including a higher utilization of mechanical ventilation (87.8% vs. 77.3%), vasoactive drugs (48.4% vs. 33.5%), and CRRT (13.2% vs. 3.3%). Conversely, the use of statins was lower in the MDR group (25.0% vs. 35.8%). In terms of clinical outcomes, the MDR group experienced significantly worse prognoses. They had a longer length of stay and higher mortality (all p < 0.001).

The original training cohort consisted of 33951 patients before resampling and was used for data preprocessing, baseline analysis, and feature selection. After the final predictors were identified, random undersampling was applied to the training set (only the final predictors included) to address class imbalance for model development. The resulting balanced training dataset included 1,893 non-MDR patients and 1,893 MDR patients. The baseline characteristics of this undersampled training cohort are presented in **Supplementary Table 5**.

Similarly, the temporal validation cohort from the MIMIC-III database (n=38583) showed highly consistent baseline patterns (**Supplementary Table 6**). The MDR group (n=4393, 11.39%) in MIMIC-III also exhibited significantly higher disease severity, a greater burden of comorbidities, and worse clinical outcomes compared to the non-MDR group (all p < 0.001).

### 3.2 Feature selection

**Figure 2:**
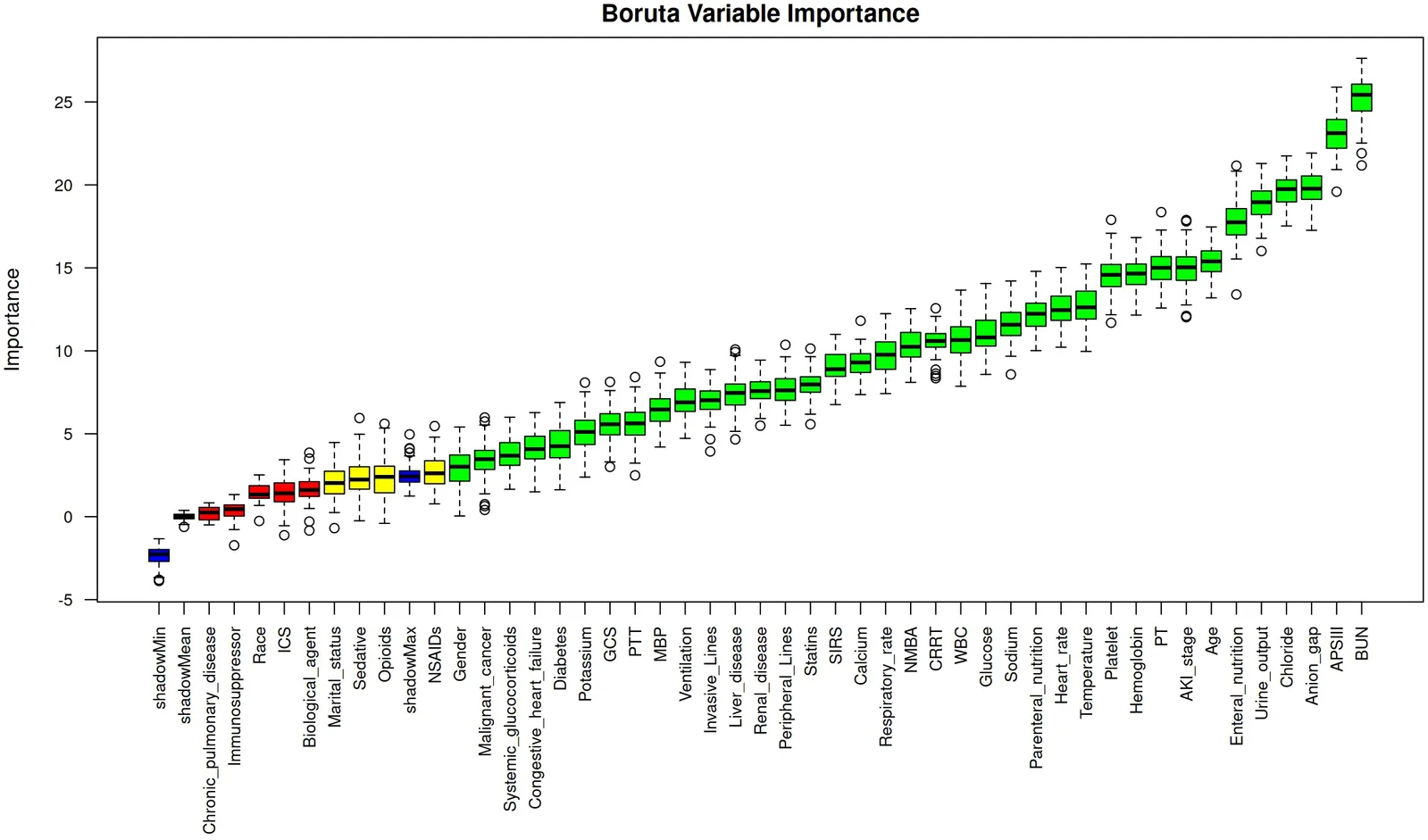
Feature importance ranking based on the Boruta algorithm for prediction of multidrug-resistant (MDR) infections in the training cohort. AKI, acute kidney injury; APSIII, Acute Physiology Score III; BUN, blood urea nitrogen; CRRT, continuous renal replacement therapy; GCS, Glasgow Coma Scale; ICS, inhaled corticosteroids; MBP, mean blood pressure; NMBA, neuromuscular blocking agent; NSAIDs, non-steroidal anti-inflammatory drugs; PT, prothrombin time; PTT, partial thromboplastin time; SIRS, Systemic Inflammatory Response Syndrome; WBC, white blood cell count. For all other abbreviations, please refer to the Glossary List.

**Figure 3:**
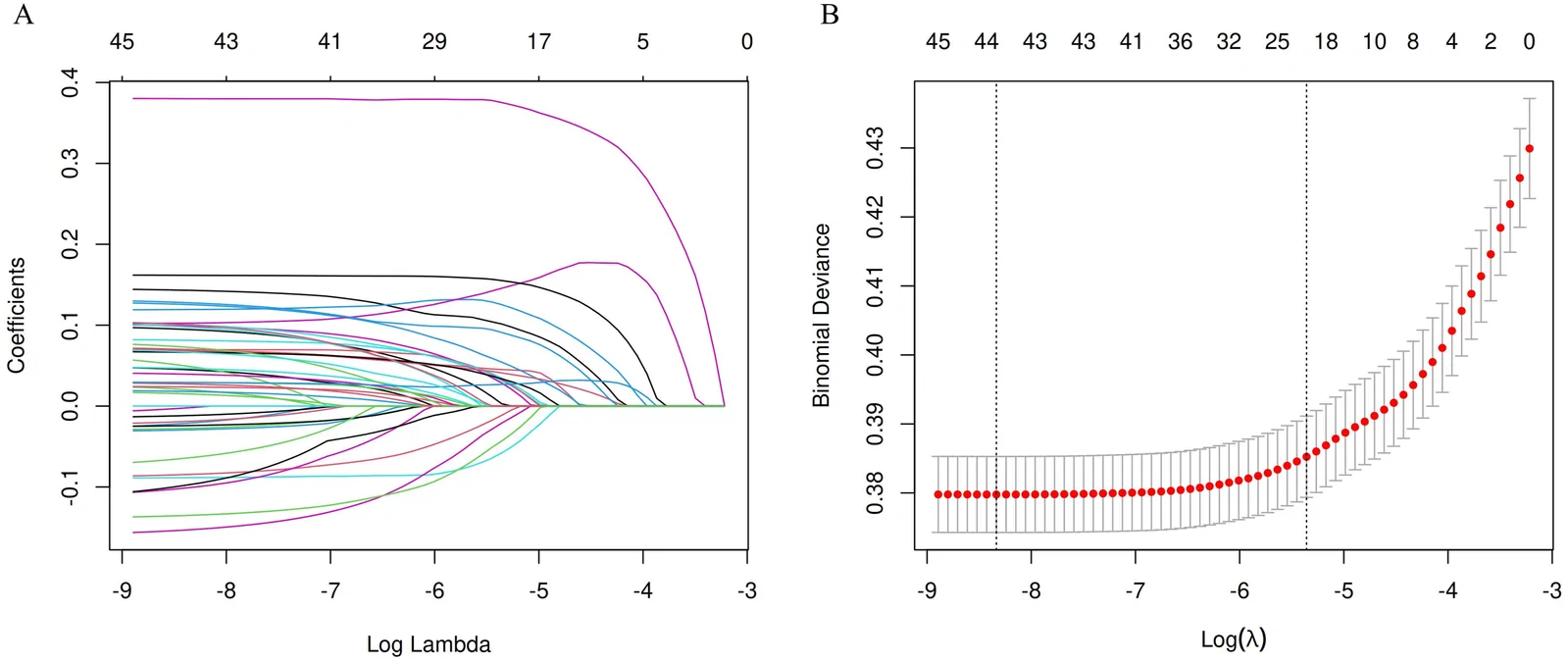
Feature selection using LASSO regression. **(A)** Coefficient profiles of candidate predictors across log(λ). **(B)** Ten-fold cross-validated binomial deviance for selecting the optimal λ. Dotted lines indicate λmin (left) and λ1se (right).

After excluding clinical outcome variables and removing highly correlated features based on the correlation analysis, the remaining variables were subjected to feature selection algorithms. The Boruta algorithm (**Figure 2**) and LASSO regression analysis (**Figure 3**) were utilized to screen for significant predictors. To ensure the stability of selected features, LASSO was combined with a 100-iteration bootstrap procedure (variables with selection probability >50% at λ.1se retained), and Boruta was independently repeated under five different random seeds (variables confirmed in ≥80% of runs retained). The final predictor set was determined by intersecting the stable features from both methods (**Supplementary Figure 4**). By intersecting the potential predictors identified by both methods, a total of 19 robust features were selected for model construction. The final set of included predictors comprised gender, temperature, heart rate, hemoglobin, platelet, BUN, liver disease, diabetes, AKI stage, SIRS, APSIII, NMBA, statins, systemic glucocorticoids, CRRT, enteral nutrition, parenteral nutrition, peripheral lines, and opioids.

### 3.3 Model performance comparison

**Figure 4:**
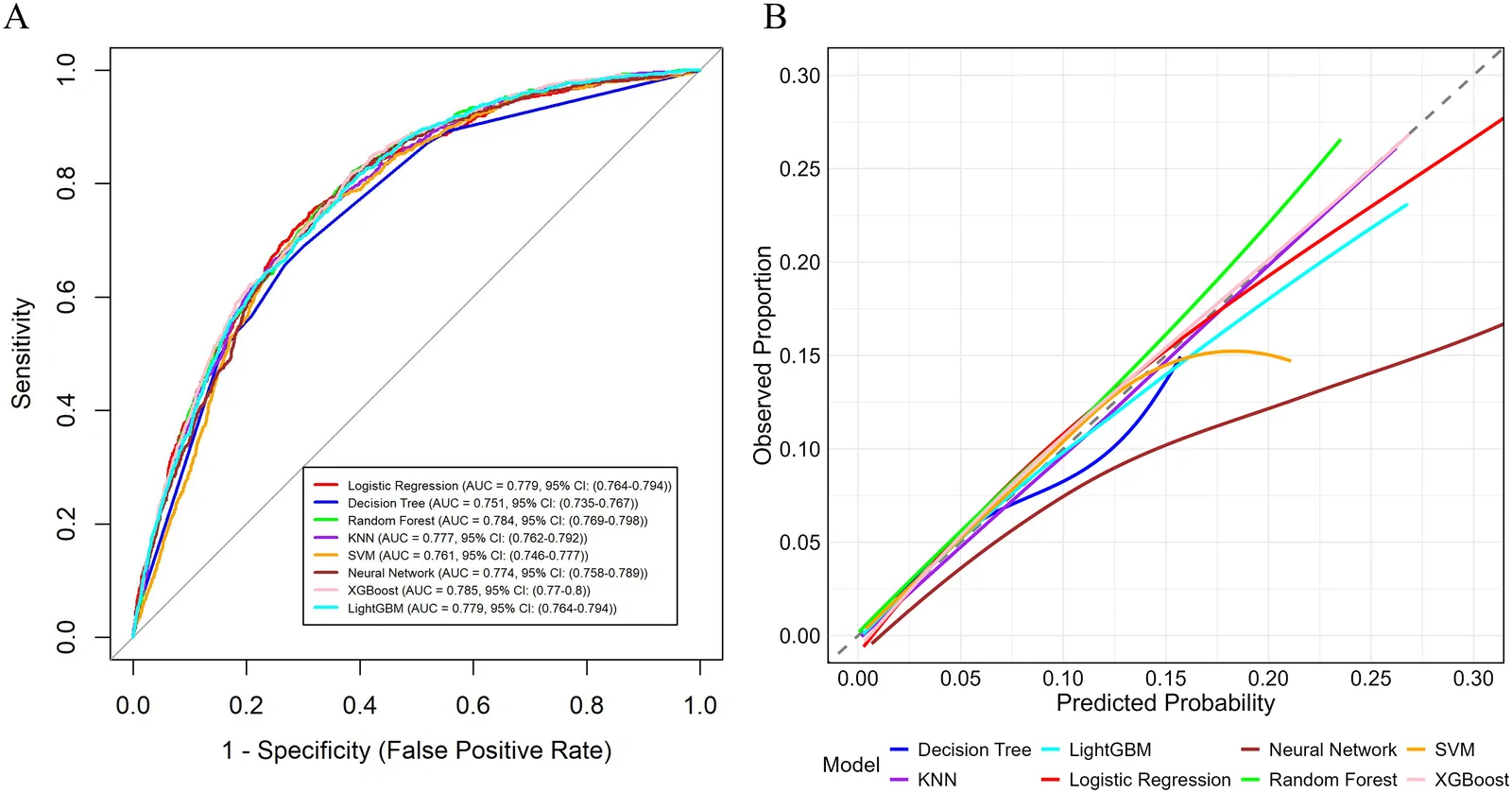
Discrimination and calibration of eight prediction models for multidrug-resistant (MDR) infections. **(A)** Receiver operating characteristic (ROC) curves comparing the discriminative performance of Logistic Regression, K-Nearest Neighbors, Decision Tree, Random Forest, Extreme Gradient Boosting, Light Gradient Boosting Machine, Support Vector Machine, and Neural Network. **(B)** Calibration curves showing agreement between predicted probability and observed probability for each model. Predicted probabilities were prevalence-recalibrated prior to calibration assessment. AUC, area under the receiver operating characteristic curve; DT, Decision Tree; KNN, K-Nearest Neighbors; LightGBM, Light Gradient Boosting Machine; LR, Logistic Regression; MDR, multidrug-resistant; NNet, Neural Network; ROC, receiver operating characteristic; RF, Random Forest; SVM, Support Vector Machine; XGBoost, Extreme Gradient Boosting.

**Figure 5:**
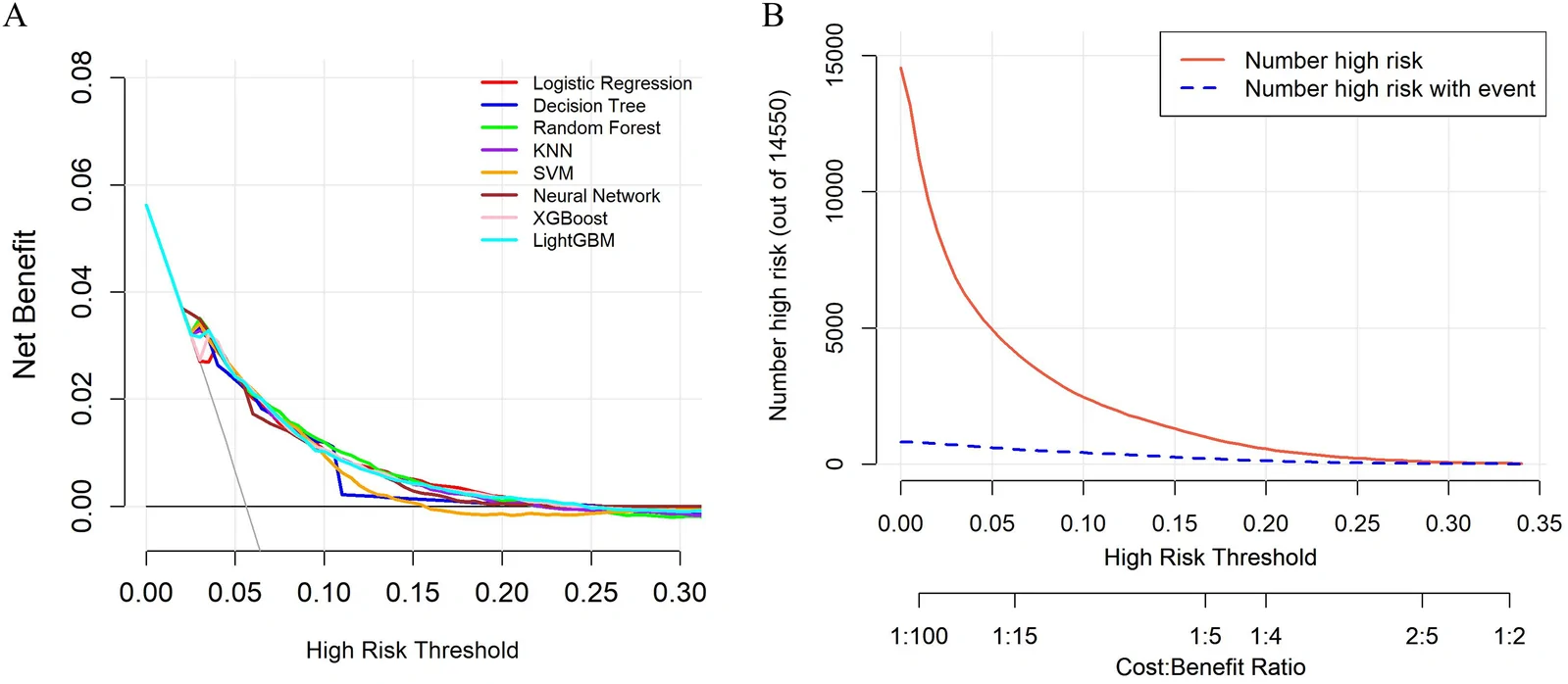
Decision-analytic evaluation of model utility. **(A)** Decision curve analysis (DCA) comparing the net benefit of eight prediction models, derived from prevalence-recalibrated probabilities, across a range of clinically relevant threshold probabilities. The gray reference lines indicate default strategies (treat-none and treat-all). **(B)** Clinical impact curve (CIC) for the Random Forest model showing the number of patients classified as high risk (solid line) and the corresponding number of true MDR events among those labeled high risk (dashed line) at each threshold. CIC, clinical impact curve; DCA, decision curve analysis; MDR, multidrug-resistant; XGBoost, Extreme Gradient Boosting.

Eight ML-based models were developed using the 19 selected predictors to estimate MDR infection risk, and their performance in the internal validation set is summarized in **Table 2**. In terms of discrimination, XGBoost and RF achieved the highest area under the ROC curve (AUC 0.785, 95% CI 0.770-0.800 and 0.784, 95% CI 0.769-0.798, respectively), followed by LR and LightGBM (both AUC 0.779). The ROC curves for all models are shown in **Figure 4A**. Regarding AUPRC, LR ranked highest (0.178, 95% CI 0.162-0.199), followed by XGBoost (0.171, 95% CI 0.156-0.188), KNN (0.168, 95% CI 0.151-0.186), and LightGBM (0.167, 95% CI 0.152-0.187), with RF (0.164, 95% CI 0.150-0.181) performing comparably. Additionally, RF achieved the highest sensitivity (0.819, 95% CI 0.793-0.845), accompanied by a correspondingly lower specificity (0.616, 95% CI 0.608-0.624), while DT achieved the highest specificity (0.697, 95% CI 0.689-0.705). LR provided the highest F1-score (0.213, 95% CI 0.198-0.228), the highest MCC (0.212, 95% CI 0.195-0.229), and the highest Cohen’s Kappa (0.129, 95% CI 0.117-0.141), while RF and LR were tied for the highest balanced accuracy (both 0.718). Calibration curves are presented in **Figure 4B**. RF demonstrated the most favorable overall probabilistic accuracy among all models, achieving the lowest Log Loss (0.1882, 95% CI 0.1788-0.1980) and the lowest Brier score (0.0497, 95% CI 0.0469-0.0527). Although the Hosmer-Lemeshow test indicated statistically significant miscalibration for all eight models (all p < 0.05), RF showed the highest (least significant) p-value (0.046) among them, together with a relatively low ECE (0.0057, 95% CI 0.0026-0.0096), whereas SVM (ECE 0.0035) and LightGBM (ECE 0.0036) achieved the lowest ECE values. NNet showed the poorest calibration, with a markedly higher ECE (0.0340) and Log Loss (0.2002) than the other models. Taken together, RF provided the most favorable overall combination of discrimination, probabilistic accuracy, and calibration among the eight models. Furthermore, DCA was performed to evaluate clinical utility (**Figure 5A**). The results indicated that all top-performing models (RF, XGBoost, LR, LightGBM, and NNet) provided a comparable net benefit over the “treat-all” or “treat-none” strategies across a range of clinically relevant threshold probabilities, with largely overlapping curves. The CIC of the RF model (**Figure 5B**) visually confirmed that the predicted high-risk population closely matched the actual number of high-risk events at higher thresholds, supporting the practical applicability of the RF model for bedside risk stratification. Considering the optimal combination of discrimination, calibration, probabilistic accuracy, and clinical utility, the RF model was selected as the final best-performing model for subsequent temporal validation and interpretability analysis. Notably, when the same eight models were additionally evaluated in an undersampled, class-balanced version of the internal validation set (**Supplementary Table 7**; **Supplementary Figures 5**, **6**), RF was consistently identified as the best-performing model across both the balanced and imbalanced validation sets.

**Table 2:** Performance comparison of eight prediction models for multidrug-resistant (MDR) infections.

Model	AUC	AUPRC	Sensitivity	Specificity	F1	Balanced accuracy	MCC	Kappa	Log Loss	Brier	ECE	HLP-value
LR	0.779 (0.764-0.794)	0.178 (0.162-0.199)	0.751 (0.722-0.781)	0.685 (0.677-0.692)	0.213 (0.198-0.228)	0.718 (0.702-0.732)	0.212 (0.195-0.229)	0.129 (0.117-0.141)	0.1898 (0.1810-0.1995)	0.0503 (0.0476-0.0533)	0.0066 (0.0053-0.0107)	<0.001
KNN	0.777 (0.762-0.792)	0.168 (0.151-0.186)	0.758 (0.729-0.786)	0.661 (0.653-0.669)	0.204 (0.190-0.216)	0.710 (0.694-0.724)	0.201 (0.185-0.216)	0.118 (0.107-0.128)	0.1897 (0.1811-0.1990)	0.0499 (0.0472-0.0529)	0.0045 (0.0029-0.0086)	0.025
DT	0.751 (0.735-0.767)	0.146 (0.134-0.160)	0.692 (0.660-0.723)	0.697 (0.689-0.705)	0.204 (0.189-0.218)	0.694 (0.678-0.710)	0.191 (0.174-0.207)	0.120 (0.107-0.132)	0.1943 (0.1850-0.2038)	0.0505 (0.0476-0.0535)	0.0047 (0.0027-0.0082)	<0.001
RF	0.784 (0.769-0.798)	0.164 (0.150-0.181)	0.819 (0.793-0.845)	0.616 (0.608-0.624)	0.198 (0.184-0.212)	0.718 (0.703-0.731)	0.204 (0.189-0.218)	0.110 (0.100-0.120)	0.1882 (0.1788-0.1980)	0.0497 (0.0469-0.0527)	0.0057 (0.0026-0.0096)	0.046
XGBoost	0.785 (0.770-0.800)	0.171 (0.156-0.188)	0.818 (0.793-0.845)	0.615 (0.608-0.624)	0.198 (0.185-0.211)	0.717 (0.703-0.731)	0.203 (0.189-0.218)	0.110 (0.100-0.119)	0.1884 (0.1792-0.1979)	0.0499 (0.0471-0.0528)	0.0042 (0.0029-0.0082)	<0.001
LightGBM	0.779 (0.764-0.794)	0.167 (0.152-0.187)	0.800 (0.772-0.824)	0.620 (0.612-0.628)	0.195 (0.182-0.207)	0.710 (0.695-0.723)	0.197 (0.181-0.210)	0.107 (0.097-0.117)	0.1895 (0.1804-0.1989)	0.0500 (0.0471-0.0529)	0.0036 (0.0025-0.0073)	<0.001
SVM	0.761 (0.746-0.777)	0.138 (0.127-0.152)	0.731 (0.701-0.761)	0.692 (0.684-0.700)	0.212 (0.197-0.226)	0.711 (0.696-0.727)	0.207 (0.190-0.223)	0.128 (0.116-0.140)	0.1933 (0.1842-0.2032)	0.0505 (0.0477-0.0535)	0.0035 (0.0023-0.0073)	<0.001
Nnet	0.774 (0.758-0.789)	0.159 (0.145-0.176)	0.802 (0.774-0.830)	0.627 (0.618-0.635)	0.199 (0.187-0.212)	0.715 (0.700-0.729)	0.202 (0.187-0.217)	0.112 (0.102-0.122)	0.2002 (0.1928-0.2081)	0.0527 (0.0502-0.0554)	0.0340 (0.0302-0.0376)	<0.001

The AUPRC was employed to assess model performance under class imbalance, while MCC and Kappa were provided as robust measures of classification agreement between predictions and actual outcomes. To evaluate model calibration, the HL p-value (Hosmer-Lemeshow test) was used, where a value > 0.05 indicates acceptable calibration; ECE, Log Loss, and Brier score were further utilized to quantify the probabilistic accuracy, with lower values representing superior alignment between predicted probabilities and observed frequencies. AUC, area under the receiver operating characteristic curve; AUPRC, area under the precision-recall curve; Brier, Brier score; DT, Decision Tree; ECE, expected calibration error; HL p-value, Hosmer-Lemeshow p-value; Kappa, Cohen’s kappa coefficient; KNN, K-Nearest Neighbors; LightGBM, Light Gradient Boosting Machine; LR, Logistic Regression; MCC, Matthews correlation coefficient; Nnet, Neural Network; RF, Random Forest; SVM, Support Vector Machine; XGBoost, Extreme Gradient Boosting.

**Figure 6:**
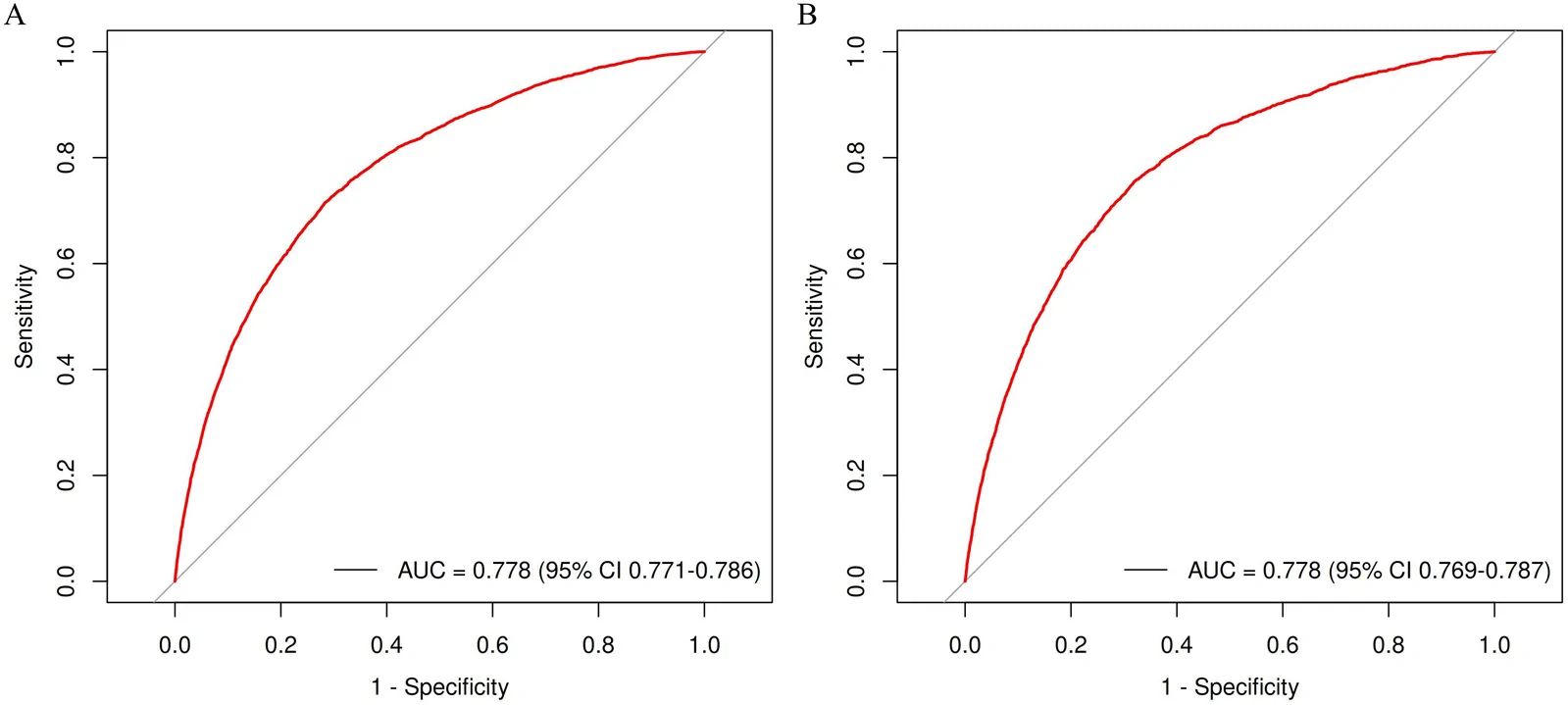
Temporal validation of the RF model in the MIMIC-III cohort. **(A)** Temporal validation through all patients in MIMIC-III cohort. **(B)** Temporal validation through selected patients in MIMIC-III cohort which didn’t overlap with those in MIMIC-IV cohort. Receiver operating characteristic (ROC) curve showing the discriminative performance of the final RF-based multidrug-resistant (MDR) infections risk model when applied to the independent MIMIC-III dataset. AUC, area under the receiver operating characteristic curve; CI, confidence interval; MDR, multidrug-resistant; MIMIC, Medical Information Mart for Intensive Care; ROC, receiver operating characteristic; RF, Random Forest.

To further assess the generalizability and robustness of the RF model, a temporal validation was performed using the independent MIMIC-III dataset. The model maintained stable performance in this temporal cohort, achieving an AUC of 0.778 (95% CI: 0.771-0.786), as illustrated in **Figure 6A**. Notably, since MIMIC-III and MIMIC-IV originated from the same institution and share a temporal overlap period, a subset of patients may appear in both databases. MIMIC-IV exclusively contains records from the MetaVision bedside charting system, whereas MIMIC-III additionally contains records from the older CareVue system. Therefore, we performed a separate temporal validation in the CareVue subset of MIMIC-III as a temporal validation. In this non-overlapping subgroup, the model achieved an AUC of 0.778 (95% CI: 0.769-0.787) (**Figure 6B**). These findings support the temporal robustness of the model to some extent. However, because both MIMIC-III and MIMIC-IV were derived from the same institution, these results should be interpreted as evidence of temporal validity, rather than full external validity across different hospitals or healthcare systems.

To examine whether model performance varied across clinically important pathogen categories, we performed exploratory pathogen-specific MDR analyses in which the positive class was restricted to each pathogen-specific MDR subgroup, while the negative class remained the original non-MDR group. These analyses were not intended to compare resistant versus susceptible isolates within the same pathogen species, but rather to assess whether the overall MDR prediction model retained discrimination for selected MDR phenotypes in the full cohort. The pathogen-specific MDR groups included carbapenem-resistant organisms (n = 144/45,934), *Klebsiella* spp. (n = 182/45,972), and *Pseudomonas* spp. (n = 150/45,940) (**Supplementary Table 8**). The RF model achieved an AUC of 0.858 (95% CI 0.833–0.882) in carbapenem-resistant organism infections, 0.835 (95% CI 0.809–0.861) in *Pseudomonas* spp. infections, and 0.822 (95% CI 0.797–0.846) in *Klebsiella* spp. infections. The model demonstrated consistent and satisfactory discriminative performance across all three pathogen-specific MDR subgroups (**Supplementary Figure 7**). Considering that overlap between the development cohort and pathogen-specific MDR subgroup cohort might lead to optimistic estimates, we further performed pathogen-specific MDR analyses in the internal validation set. The distribution of MDR status across microbiological subgroups was shown in **Supplementary Table 9**. The model remained discriminative, with AUCs of 0.849 (95% CI 0.794–0.904) for carbapenem-resistant organism infections, 0.823 (95% CI 0.779–0.867) for Klebsiella spp. infections, and 0.870 (95% CI 0.825–0.916) for Pseudomonas spp. infections (**Supplementary Figure 8**). These findings suggest that the RF model maintained good discrimination across clinically relevant MDR pathogen subgroups, although these estimates should be interpreted cautiously because of the limited number of positive cases. As a sensitivity analysis, we restricted the evaluation cohort to patients whose MDR status could be determined within the prespecified time window. Specifically, for each window (48 hours and 72 hours after ICU admission), we excluded patients with ICU stay shorter than the corresponding window and those whose first MDR detection occurred after that time point. The original final model was then re-evaluated in the resulting restricted cohort. The AUCs of 48-hour and 72-hour time windows after ICU admission were 0.741 (95% CI 0.713–0.768) and 0.722 (95% CI 0.692–0.751), respectively (**Supplementary Figure 9**). The AUC was slightly lower than that in the primary analysis, which may reflect the difference in outcome definition between the primary and sensitivity analyses.

### 3.4 Interpretability of the machine learning model using SHAP

Although the RF model demonstrated superior predictive performance, its complex “black box” nature challenges clinical interpretability. To address this, SHAP analysis was employed to quantify the contribution of each feature to the prediction of MDR infections.

**Figure 7:**
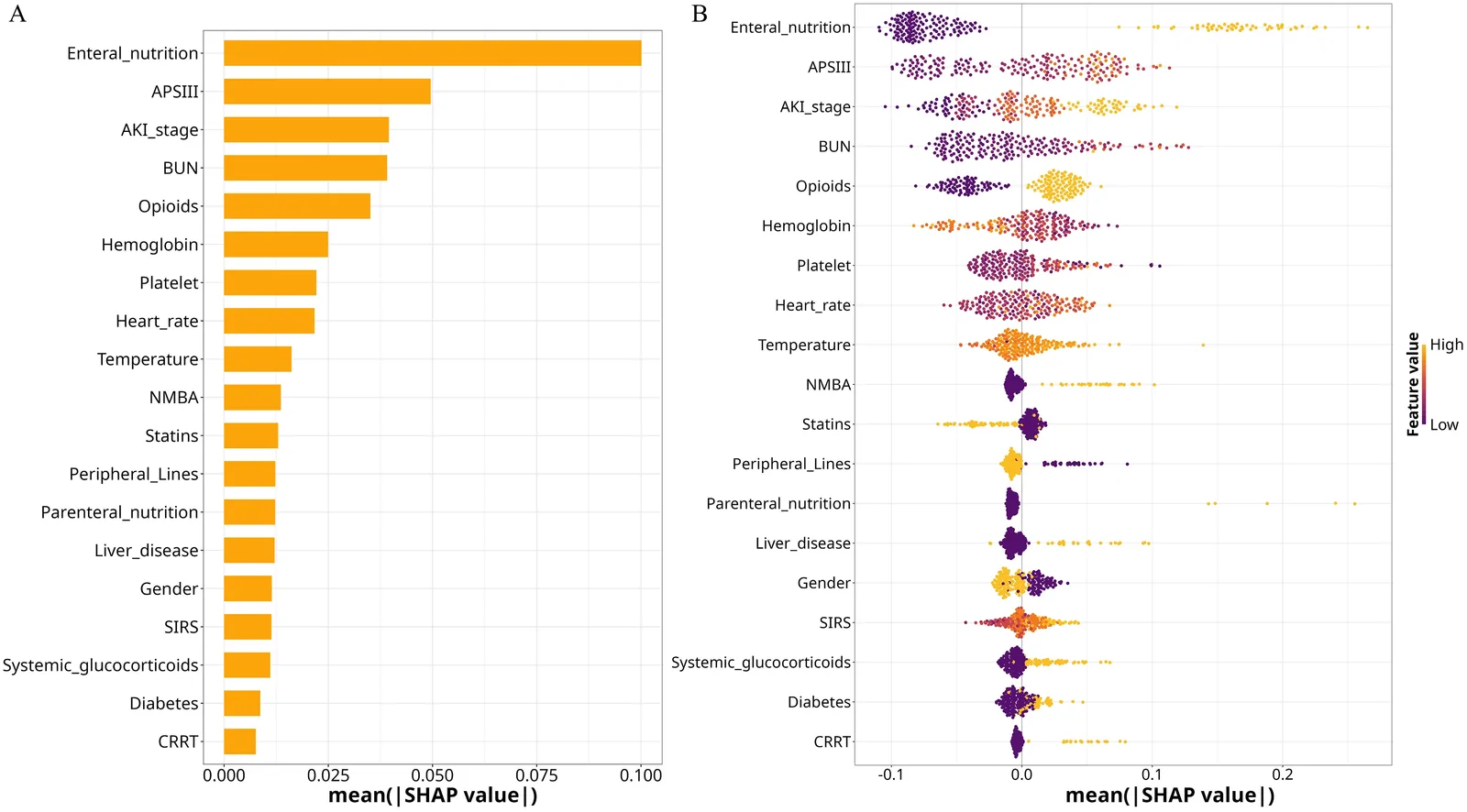
SHAP-based global interpretability of the best-performing RF model for early MDR infections risk prediction. **(A)** Global feature importance ranked by the mean absolute SHAP value, reflecting each predictor’s overall contribution to the model output. **(B)** SHAP summary (beeswarm) plot showing the distribution and direction of feature effects. Each point represents one patient, with the x-axis indicating the SHAP value (positive values increase the predicted risk of MDR infections, negative values decrease it). Point color denotes the feature magnitude (high vs low), enabling visualization of how higher or lower values of each predictor shift the model prediction. AKI, acute kidney injury; APSIII, Acute Physiology Score III; BUN, blood urea nitrogen; CRRT, continuous renal replacement therapy; NMBA, neuromuscular blocking agents; RF, Random Forest; SHAP, SHapley Additive exPlanations; SIRS, systemic inflammatory response syndrome.

**Figure 7A** displays the ranking of feature importance based on the mean absolute SHAP values. Enteral nutrition, APSIII, AKI stage, BUN, opioids use, hemoglobin, and platelet were identified as the leading influential predictors. The SHAP beeswarm plot (**Figure 7B**) further elucidates the directional relationship between features and MDR infection risk. Each dot represents a single patient. High values of APSIII, AKI stage, BUN, and enteral nutrition were distributed on the positive side of the x-axis, suggesting that greater disease severity, renal dysfunction, and enteral nutrition use are positively correlated with an increased risk of MDR infections. Similarly, the use of NMBA, peripheral lines, parenteral nutrition, and the presence of liver disease and SIRS were also associated with positive SHAP values, indicating an elevated risk. Conversely, higher values of hemoglobin, platelet, and statin use were associated with negative SHAP values. The detailed marginal effects of continuous features were displayed through SHAP dependence plots (**Supplementary Figure 10**).

**Figure 8:**
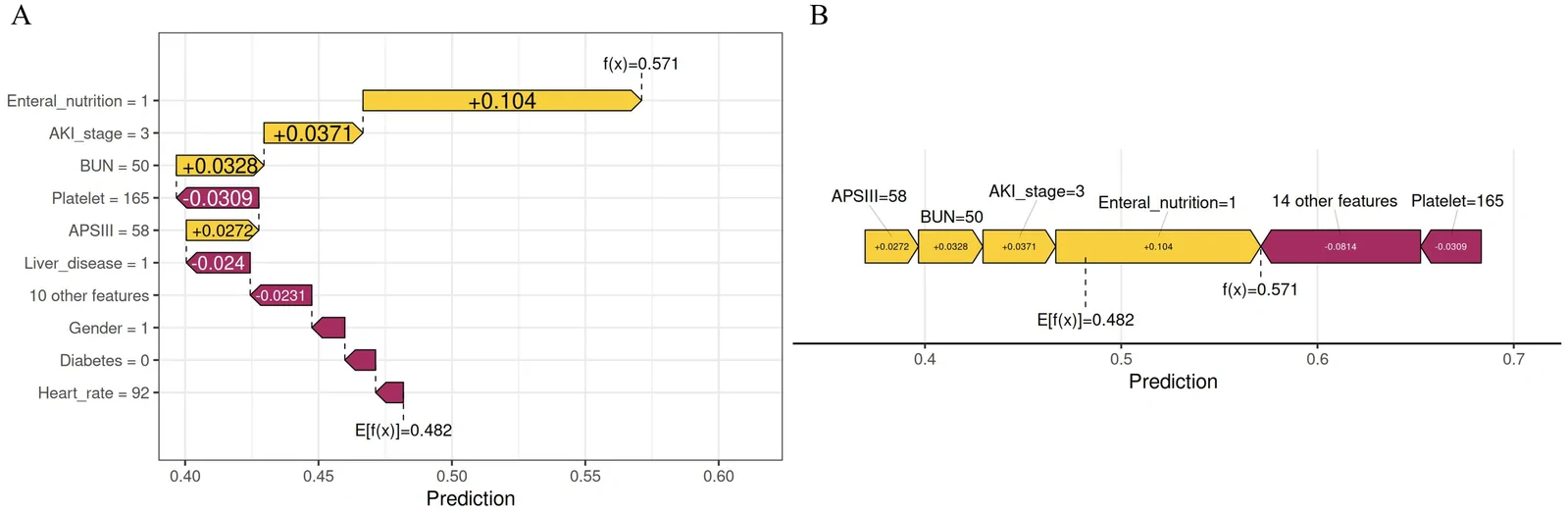
Individual-level SHAP explanation of the best-performing RF model based on the uncalibrated model output. **(A)** SHAP waterfall plot for a representative patient illustrating how the prediction is formed by sequentially adding feature contributions to the baseline model output. Features with positive SHAP values shift the prediction toward a higher MDR infections risk, whereas negative SHAP values shift it toward a lower risk. The plot starts from the expected model output and ends at the patient-specific predicted probability. **(B)** SHAP force plot depicting the same case, where features pushing the prediction upward and downward are displayed on opposite sides of the baseline, highlighting the net shift to the final individualized uncalibrated model output. AKI, acute kidney injury; APSIII, Acute Physiology Score III; BUN, blood urea nitrogen; MDR, multidrug-resistant; RF, Random Forest; SHAP, SHapley Additive exPlanations.

Furthermore, SHAP allows for personalized interpretation of predictions for individual patients. **Figure 8** visualizes the decision-making process for a randomly selected patient using a waterfall plot (**Figure 8A**) and a force plot (**Figure 8B**). For this specific individual, the presence of enteral nutrition (= 1), AKI stage 3, elevated BUN (= 50), and APSIII (= 58) pushed the prediction towards a higher risk (positive contribution), whereas platelet count (= 165) and liver disease (= 1) did the opposite, lowering the final predicted probability. Starting from the baseline expected value of 0.482, these combined contributions yielded a final prediction of 0.571. This demonstrates the model’s capability to provide clinically actionable, patient-specific risk profiles.

### 3.5 Development of a web-based risk calculator

**Figure 9:**
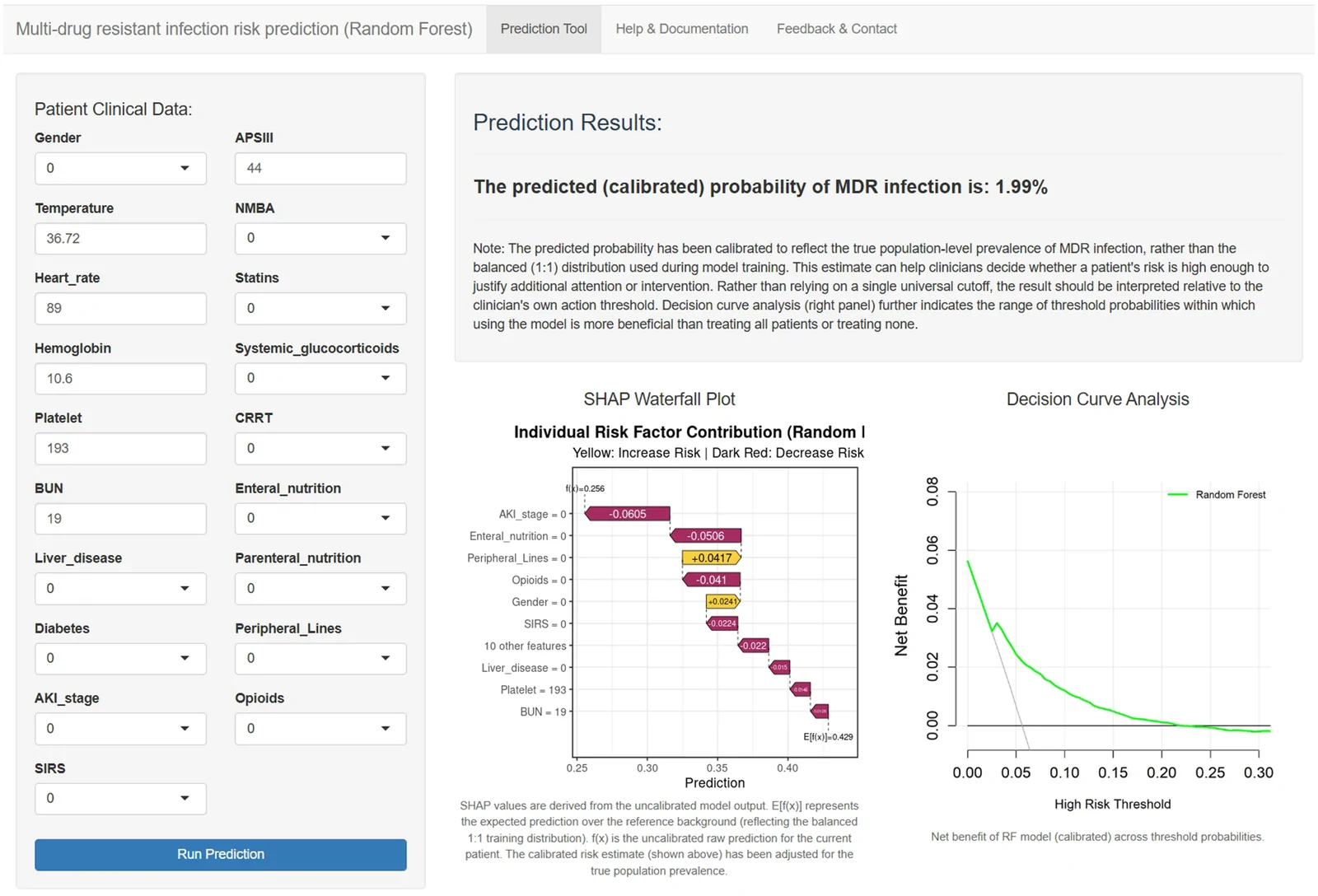
Web-based MDR infections risk calculator implemented with the best-performing RF model using R Shiny. The left panel provides structured input fields for the 19 predictors included in the final model, with explicit coding instructions for binary and categorical variables. After submission, the application returns the individualized predicted probability of MDR infection in real time and provides a brief note to guide interpretation of the predicted risk relative to the clinician’s action threshold. To improve transparency, the right panel simultaneously displays a patient-specific SHAP waterfall plot, visualizing the direction and magnitude of each feature’s contribution to the uncalibrated model output, together with the decision curve analysis (DCA) of the Random Forest model to illustrate its potential clinical utility across threshold probabilities. MDR, multidrug-resistant; RF, Random Forest; SHAP, SHapley Additive exPlanations; DCA, decision curve analysis; AKI, acute kidney injury; APSIII, Acute Physiology Score III; BUN, blood urea nitrogen; SIRS, systemic inflammatory response syndrome; NMBA, neuromuscular blocking agents; CRRT, continuous renal replacement therapy.

To facilitate the translation of the RF model into clinical practice, a user-friendly web-based calculator was developed using the Shiny package in R software (https://predic2.shinyapps.io/ICU_MDR/). As shown in **Figure 9**, the interface allows clinicians to enter the values of the 19 predictors required by the final model, with explicit instructions for binary and categorical coding to improve usability. After submission, the application immediately returns the individualized predicted probability of MDR infection. To support interpretation, the interface also provides a brief explanatory note indicating that the predicted probability should be understood as a continuous risk estimate and interpreted relative to the clinician’s action threshold, rather than as a fixed binary classification. In addition, to enhance transparency and trust in the AI-driven prediction, the application generates a patient-specific SHAP waterfall plot showing how each feature contributes to increasing or decreasing the predicted risk. The calculator also presents the decision curve analysis of the RF model, allowing users to appreciate the threshold probability range in which model-guided decision-making may provide net clinical benefit. Together, this interactive tool supports personalized risk assessment, interpretable prediction, and clinically informed decision-making.

## 4 Discussion

This study provides an interpretable framework for early risk stratification of multidrug-resistant infections in ICU patients using routinely available admission data. By combining temporal validation, SHapley Additive exPlanations, and a deployable web-based calculator, the work targets a practical plight in MDR prediction, that clinicians need actionable risk estimates before microbiology and susceptibility results become available.

Prediction of MDR infections is not a new topic, and several predictive models have been proposed. However, some existing tools were developed for specific scenarios or subpopulations, such as ventilator-associated pneumonia cohorts or neuro-ICU/neurosurgical patients, so their applicability to an unselected ICU population remains uncertain (Jiang et al., 2023; Cui et al., 2025; Hu et al., 2025). In addition, a proportion of models were derived from single-center datasets with limited sample size and without independent external validation, making generalizability difficult to judge (Hu et al., 2025; Zhao et al., 2025). Even when external validation was conducted, performance sometimes declined in independent cohorts, suggesting limited transportability across settings (Li et al., 2024). Moreover, deep learning-based approaches may yield acceptable discrimination but often function as “black boxes”, limiting clinical interpretability and reducing confidence in bedside use (Wang et al., 2023). Finally, current studies stop at model development and reporting, without providing a deployable calculator or interface that allows clinicians to directly estimate individual risk. In contrast, our model was developed for an unselected ICU population using a large ICU cohort, underwent independent temporal validation, demonstrated stable performance across cohorts, provided transparent SHAP-based explanations to mitigate “black-box” concerns, and was implemented as a deployable web-based calculator to enable direct bedside risk estimation.

Enteral nutrition ranked highest in global SHAP importance in our model. It is essential to recognize that SHAP values quantify each feature’s marginal contribution to model predictions relative to a reference baseline, and they do not establish causal relationships between clinical exposures and outcomes. Accordingly, the prominent position of enteral nutrition in our SHAP analysis should emphatically not be interpreted as evidence that enteral nutrition itself causes or promotes MDR infections. On the contrary, enteral nutrition is generally favored in critical care because it improves state of nutrition, helps maintain gut integrity and immune function (Schörghuber and Fruhwald, 2018). We propose that the dominant SHAP contribution of enteral nutrition in our model is most plausibly explained by profound confounding by indication. In ICU clinical practice, enteral nutrition is preferentially initiated in patients who are more severely ill, mechanically ventilated, and anticipated to require prolonged critical care support. Beyond confounding, several procedural mechanisms might theoretically modulate infection-related risks associated with enteral feeding in specific clinical circumstances, most notably, the potential for microaspiration of gastric or small-bowel contents via the enteral tube in mechanically ventilated patients (Drakulovic et al., 1999; Metheny et al., 2006), and the risk of contamination during formula preparation or administration (Baik et al., 2025). Clinicians and policymakers should therefore interpret enteral nutrition’s prominence in our model as a reflection of illness severity and high-intensity ICU care requirements, not as a signal to modify established enteral nutrition practices.

Beyond enteral nutrition, elevated BUN, opioid use, and higher AKI stage each demonstrated notable SHAP contributions. Elevated BUN and advanced AKI stage are associated with accumulation of uremic solutes that can impair innate immune responses and necessitate renal replacement therapy with attendant invasive access (Cohen and Hörl, 2012). Opioid use, beyond reflecting sedation requirements in ventilated patients, carries well-documented immunomodulatory effects that may independently compromise host defenses (Plein and Rittner, 2018). At the same time, all three variables are associated with overall illness severity and treatment intensity, which reflects confounding by indication.

Statin use tended to contribute negative SHAP values, indicating an association with a lower predicted risk of MDR infections. This pattern is biologically plausible because statins have well-described pleiotropic effects beyond lipid lowering, including anti-inflammatory, antioxidative, and immunomodulatory actions that may attenuate dysregulated host responses in critical illness and potentially reduce susceptibility to secondary infections (Mahmoudi et al., 2026). In parallel, a growing body of experimental and translational literature suggests that certain statins may exert direct or indirect anti-infective effects, such as interfering with bacterial membrane microdomains, modulating virulence-related pathways, or enhancing antibiotic activity in selected settings, that have led to the idea of statins as potential “antimicrobial resistance breakers” (2017). However, the evidence remains heterogeneous, and some reviews emphasize that statins could also behave as markers rather than true “breakers”, highlighting uncertainty around the magnitude and consistency of clinically meaningful antimicrobial effects (Ko et al., 2017; Abavisani et al., 2025). Therefore, in our cohort, the statin signal should be interpreted conservatively. It may reflect a combination of host-modulating pleiotropic effects, possible adjunct anti-infective properties, and residual confounding, rather than a definitive causal protection against MDR infections.

Our model provides promising potential for early MDR risk stratification at ICU admission using routinely available variables. By identifying patients with a high predicted probability of MDR infections during the initial “blind window” before culture and susceptibility results return, the tool can support more targeted empirical antimicrobial decisions, prompting timely broad-spectrum or anti-MDR coverage, while helping avoid unnecessary escalation in low-risk patients to strengthen antimicrobial stewardship. In parallel, high-risk flags can guide infections-prevention workflows, such as prioritizing isolation precautions, reinforcing catheter and feeding-tube care bundles, and closer surveillance for early clinical deterioration. The Shiny-based web calculator enables rapid deployment and standardized use across shifts and providers, facilitating consistent decision-making, communication within the ICU team, and resource allocation (e.g., microbiology sampling priority and infections-control attention) for patients most likely to harbor or develop MDR infections.

Several limitations should be acknowledged. First, as a retrospective observational analysis based on the MIMIC databases, residual confounding and bias are unavoidable. Important determinants of multidrug-resistant risk, including pre-ICU antibiotic exposure, prior healthcare contact, baseline colonization, and inter-facility transfer, are incompletely captured. Therefore, predictors identified by the model should be interpreted primarily as risk markers rather than causal drivers. Additionally, because the primary outcome was defined over the remainder of the ICU stay, patients with longer ICU stays may have had greater opportunity for microbiological sampling and MDR detection. Therefore, part of the model signal may reflect differential follow-up duration and testing opportunity in addition to baseline MDR risk. Second, although we performed temporal validation using MIMIC-III, this database and MIMIC-IV originate from the same healthcare system. Therefore, our validation cohort was not fully independent in terms of institution, region, or practice environment. The MIMIC-III validation, especially the CareVue subset containing predominantly pre-2008 records, should be considered a temporal validation rather than a strict multi-center external validation. Consequently, the generalizability of the model to other hospitals, healthcare systems, and geographic regions still requires further confirmation in future multicenter studies. Third, although subgroup validation demonstrated consistent model performance across *Pseudomonas* spp., *Klebsiella* spp., and carbapenem-resistant subgroups, the sample sizes within individual microbiological subgroups were limited, precluding formal statistical comparisons of AUC across subgroups or the development of pathogen-specific prediction models. And due to the limited sample sizes, the subgroup analyses should be considered exploratory rather than confirmatory outcome. Future studies with larger, prospectively collected microbiological datasets, linking admission-level clinical features to subsequently confirmed pathogen identity and resistance phenotype, are needed to explore whether pathogen-stratified or resistance phenotype-specific models could offer incremental precision over a generalized host-centric approach. Finally, this study focused on predictive performance rather than clinical impact. Prospective studies are needed to determine whether model-guided decision-making improves outcomes, reduces inappropriate broad-spectrum antibiotic use, and is acceptable and feasible within real-world ICU workflows.

In conclusion, we developed and temporally validated an interpretable RF model for early prediction of MDR infections in ICU patients using routinely available admission data. The model demonstrated stable performance across MIMIC-IV and MIMIC-III, provided transparent SHAP-based explanations, and was implemented as a deployable web calculator to facilitate bedside risk stratification and support timely antimicrobial stewardship and resource allocation.

## Data Availability

Publicly available datasets were analyzed in this study. This data can be found here: https://physionet.org/content/mimiciv/3.1/ and https://physionet.org/content/mimiciii/1.4/.

## Glossary

AKI: Acute kidney injury
ALP: Alkaline phosphatase
ALT: Alanine aminotransferase
APSIII: Acute Physiology Score III
AST: Aspartate aminotransferase
AUC: Area under the curve
AUPRC: Area under the precision–recall curve
BUN: Blood urea nitrogen
CIC: Clinical impact curve
CRP: C-reactive protein
CRRT: Continuous renal replacement therapy
DBP: Diastolic blood pressure
DCA: Decision curve analysis
DT: Decision tree
EN: Enteral nutrition
GCS: Glasgow Coma Scale
GGT: Gamma-glutamyl transferase
ICU: Intensive care unit
ICS: Inhaled corticosteroids
INR: International normalized ratio
IQR: Interquartile range
LASSO: Least Absolute Shrinkage and Selection Operator
LDH: Lactate dehydrogenase
LightGBM: Light Gradient Boosting Machine
LODS: Logistic Organ Dysfunction System
LR: Logistic regression
MDR: Multidrug-resistant
MIMIC: Medical Information Mart for Intensive Care
MIMIC-III: Medical Information Mart for Intensive Care III
MIMIC-IV: Medical Information Mart for Intensive Care IV
ML: Machine learning
NMBA: Neuromuscular blocking agent
NSAIDs: Non-steroidal anti-inflammatory drugs
OASIS: Oxford Acute Severity of Illness Score
PCO2: Partial pressure of carbon dioxide
pH: Potential of hydrogen
PO2: Partial pressure of oxygen
PT: Prothrombin time
PTT: Partial thromboplastin time
RBC: Red blood cell
RF: Random forest
ROC: Receiver operating characteristic
SAPSII: Simplified Acute Physiology Score II
SBP: Systolic blood pressure
SD: Standard deviation
SHAP: SHapley Additive exPlanations
SIRS: Systemic Inflammatory Response Syndrome
SO2: Oxygen saturation
SOFA: Sequential Organ Failure Assessment
SQL: Structured Query Language
SVM: Support vector machine
WBC: White blood cell
XGBoost: Extreme Gradient Boosting.
