# Supplementary material for: Development and validation of an interpretable machine learning model for predicting multidrug-resistant infections in ICU patients
Source: Front Cell Infect Microbiol. 2026 Aug 10;16:1807363. doi: 10.3389/fcimb.2026.1807363 (PMC13500264; doi:10.3389/fcimb.2026.1807363)
Supplement: Supplementary file 1 [file Table1.docx]

**Supplementary Table 1** Missing data profile of candidate variables in the MIMIC-IV cohort.

| Variables | Missing count | Missing ratio |
| --- | --- | --- |
| Age | 0 | 0 |
| Gender | 0 | 0 |
| Race | 0 | 0 |
| Marital status | 0 | 0 |
| Temperature | 189 | 0.003897 |
| Respiratory rate | 39 | 0.000804 |
| MBP | 12 | 0.000247 |
| DBP | 3 | 6.19E-05 |
| SBP | 3 | 6.19E-05 |
| Heart rate | 1 | 2.06E-05 |
| Height | 47494 | 0.979238 |
| Weight | 20155 | 0.415558 |
| Urine output | 1118 | 0.023051 |
| WBC | 308 | 0.00635 |
| RBC | 308 | 0.00635 |
| Hemoglobin | 312 | 0.006433 |
| Hematocrit | 289 | 0.005959 |
| Platelet | 304 | 0.006268 |
| CRP | 46260 | 0.953795 |
| Total bilirubin | 23252 | 0.479413 |
| AST | 22935 | 0.472877 |
| ALP | 23314 | 0.480691 |
| ALT | 23107 | 0.476423 |
| GGT | 47868 | 0.986949 |
| Total protein | 46742 | 0.963733 |
| Globulin | 47699 | 0.983464 |
| Albumin | 29547 | 0.609204 |
| BUN | 280 | 0.005773 |
| Creatinine | 275 | 0.00567 |
| LDH | 29865 | 0.61576 |
| Glucose | 357 | 0.007361 |
| Sodium | 271 | 0.005588 |
| Potassium | 283 | 0.005835 |
| Calcium | 2571 | 0.053009 |
| Anion gap | 300 | 0.006185 |
| Chloride | 271 | 0.005588 |
| Fibrinogen | 32670 | 0.673594 |
| PT | 4450 | 0.091751 |
| PTT | 4656 | 0.095998 |
| INR | 4450 | 0.091751 |
| D_dimer | 47796 | 0.985464 |
| PCO2 | 16684 | 0.343993 |
| PO2 | 16681 | 0.343931 |
| SO2 | 31980 | 0.659368 |
| Base excess | 16684 | 0.343993 |
| Bicarbonate | 47182 | 0.972805 |
| PH | 16684 | 0.343993 |
| Lactate | 19974 | 0.411827 |
| Liver disease | 0 | 0 |
| Renal disease | 0 | 0 |
| Malignant cancer | 0 | 0 |
| Diabetes | 0 | 0 |
| Chronic pulmonary disease | 0 | 0 |
| Congestive heart failure | 0 | 0 |
| AKI | 0 | 0 |
| AKI stage | 0 | 0 |
| SIRS | 0 | 0 |
| OASIS | 0 | 0 |
| LODS | 0 | 0 |
| APSIII | 0 | 0 |
| GCS | 79 | 0.001629 |
| SAPSII | 0 | 0 |
| SOFA | 65 | 0.00134 |
| NMBA | 0 | 0 |
| Sedative | 0 | 0 |
| Opioids | 0 | 0 |
| NSAIDs | 0 | 0 |
| Statins | 0 | 0 |
| Systemic glucocorticoids | 0 | 0 |
| ICS | 0 | 0 |
| Vasoactive | 0 | 0 |
| Immunosuppressor | 0 | 0 |
| Biological agent | 0 | 0 |
| CRRT | 0 | 0 |
| Ventilation | 0 | 0 |
| Enteral nutrition | 0 | 0 |
| Parenteral nutrition | 0 | 0 |
| Invasive Lines | 0 | 0 |
| Peripheral Lines | 0 | 0 |
| Tubes | 0 | 0 |
| Los hospital | 0 | 0 |
| Hospital mortality | 0 | 0 |
| Los ICU | 0 | 0 |
| ICU mortality | 0 | 0 |
| ICU time 30d | 0 | 0 |
| ICU outcome 30d | 0 | 0 |
| ICU time 90d | 0 | 0 |
| ICU outcome 90d | 0 | 0 |
| ICU time 180d | 0 | 0 |
| ICU outcome 180d | 0 | 0 |

Abbreviations: AKI, acute kidney injury; ALP, alkaline phosphatase; ALT, alanine aminotransferase; APSIII, Acute Physiology Score III; AST, aspartate aminotransferase; AUC, area under the receiver operating characteristic curve; BUN, blood urea nitrogen; CIC, clinical impact curve; CRP, C-reactive protein; CRRT, continuous renal replacement therapy; DBP, diastolic blood pressure; DCA, decision curve analysis; GCS, Glasgow Coma Scale; GGT, gamma-glutamyl transferase; ICU, intensive care unit; ICS, inhaled corticosteroids; INR, international normalized ratio; LDH, lactate dehydrogenase; LODS, Logistic Organ Dysfunction System; MBP, mean blood pressure; MIMIC, Medical Information Mart for Intensive Care; NMBA, neuromuscular blocking agent; NSAIDs, non-steroidal anti-inflammatory drugs; OASIS, Oxford Acute Severity of Illness Score; PCO2, partial pressure of carbon dioxide; PH, potential of hydrogen; PO2, partial pressure of oxygen; PT, prothrombin time; PTT, partial thromboplastin time; RBC, red blood cell; SAPSII, Simplified Acute Physiology Score II; SBP, systolic blood pressure; SIRS, Systemic Inflammatory Response Syndrome; SO2, oxygen saturation; SOFA, Sequential Organ Failure Assessment; WBC, white blood cell.

**Supplementary Table 2** Missing data profile of candidate variables in the MIMIC-III cohort.

| Variables | Missing count | Missing ratio |
| --- | --- | --- |
| Age | 0 | 0 |
| Gender | 0 | 0 |
| Race | 0 | 0 |
| Marital status | 0 | 0 |
| Heart rate | 288 | 0.007464 |
| SBP | 314 | 0.008138 |
| DBP | 315 | 0.008164 |
| MBP | 293 | 0.007594 |
| Respiratory rate | 325 | 0.008423 |
| Temperature | 965 | 0.025011 |
| Weight | 2508 | 0.065003 |
| Height | 18475 | 0.478838 |
| Urine output | 1466 | 0.037996 |
| WBC | 1080 | 0.027992 |
| CRP | 38218 | 0.99054 |
| RBC | 1201 | 0.031128 |
| Hematocrit | 765 | 0.019827 |
| Hemoglobin | 1178 | 0.030532 |
| Platelet | 969 | 0.025115 |
| Total bilirubin | 25221 | 0.653682 |
| ALT | 25119 | 0.651038 |
| AST | 25129 | 0.651297 |
| ALP | 25455 | 0.659747 |
| GGT | 38417 | 0.995698 |
| Total protein | 38109 | 0.987715 |
| Albumin | 28062 | 0.727315 |
| Globulin | 38390 | 0.994998 |
| BUN | 664 | 0.01721 |
| Creatinine | 647 | 0.016769 |
| LDH | 29661 | 0.768758 |
| Potassium | 1249 | 0.032372 |
| Sodium | 956 | 0.024778 |
| Calcium | 6762 | 0.175259 |
| Chloride | 815 | 0.021123 |
| Anion gap | 1569 | 0.040666 |
| Glucose | 1650 | 0.042765 |
| INR | 7747 | 0.200788 |
| PT | 7745 | 0.200736 |
| PTT | 7880 | 0.204235 |
| D dimer | 38431 | 0.99606 |
| Fibrinogen | 31446 | 0.815022 |
| Base excess | 15481 | 0.401239 |
| Bicarbonate | 37813 | 0.980043 |
| Lactate | 21088 | 0.546562 |
| SO2 | 25243 | 0.654252 |
| PCO2 | 15483 | 0.401291 |
| PH | 14566 | 0.377524 |
| PO2 | 15483 | 0.401291 |
| Congestive heart failure | 0 | 0 |
| Chronic pulmonary disease | 0 | 0 |
| Diabetes | 0 | 0 |
| Renal disease | 0 | 0 |
| Liver disease | 0 | 0 |
| Malignant cancer | 0 | 0 |
| AKI | 0 | 0 |
| AKI stage | 0 | 0 |
| GCS | 304 | 0.007879 |
| SOFA | 0 | 0 |
| SIRS | 0 | 0 |
| APSIII | 0 | 0 |
| LODS | 0 | 0 |
| OASIS | 0 | 0 |
| SAPSII | 0 | 0 |
| Vasoactive | 0 | 0 |
| ICS | 0 | 0 |
| Systemic glucocorticoids | 0 | 0 |
| Statins | 0 | 0 |
| NSAIDs | 0 | 0 |
| Opioids | 0 | 0 |
| Sedative | 0 | 0 |
| NMBA | 0 | 0 |
| Biological agent | 0 | 0 |
| Immunosuppressor | 0 | 0 |
| CRRT | 0 | 0 |
| Ventilation | 0 | 0 |
| Enteral nutrition | 0 | 0 |
| Parenteral nutrition | 0 | 0 |
| Invasive Lines | 0 | 0 |
| Peripheral Lines | 0 | 0 |
| Los hospital | 0 | 0 |
| Hospital mortality | 0 | 0 |
| Los ICU | 0 | 0 |
| ICU mortality | 0 | 0 |
| ICU time 30d | 0 | 0 |
| ICU outcome 30d | 0 | 0 |
| ICU time 90d | 0 | 0 |
| ICU outcome 90d | 0 | 0 |
| ICU time 180d | 0 | 0 |
| ICU outcome 180d | 0 | 0 |

Abbreviations: AKI, acute kidney injury; ALP, alkaline phosphatase; ALT, alanine aminotransferase; APSIII, Acute Physiology Score III; AST, aspartate aminotransferase; AUC, area under the receiver operating characteristic curve; BUN, blood urea nitrogen; CIC, clinical impact curve; CRP, C-reactive protein; CRRT, continuous renal replacement therapy; DBP, diastolic blood pressure; DCA, decision curve analysis; GCS, Glasgow Coma Scale; GGT, gamma-glutamyl transferase; ICU, intensive care unit; ICS, inhaled corticosteroids; INR, international normalized ratio; LDH, lactate dehydrogenase; LODS, Logistic Organ Dysfunction System; MBP, mean blood pressure; MIMIC, Medical Information Mart for Intensive Care; NMBA, neuromuscular blocking agent; NSAIDs, non-steroidal anti-inflammatory drugs; OASIS, Oxford Acute Severity of Illness Score; PCO2, partial pressure of carbon dioxide; PH, potential of hydrogen; PO2, partial pressure of oxygen; PT, prothrombin time; PTT, partial thromboplastin time; RBC, red blood cell; SAPSII, Simplified Acute Physiology Score II; SBP, systolic blood pressure; SIRS, Systemic Inflammatory Response Syndrome; SO2, oxygen saturation; SOFA, Sequential Organ Failure Assessment; WBC, white blood cell.

**Supplementary Table 3** Distribution of means and coefficients of variation (CV) for clinical variables across different random seeds for missForest.

| Variable | Mean_seed1 | Mean_seed42 | Mean_seed100 | Mean_seed2023 | Mean_seed9999 | CV (%) |
| --- | --- | --- | --- | --- | --- | --- |
| Temperature | 36.38 | 36.37 | 36.38 | 36.38 | 36.38 | 0.02 |
| Respiratory rate | 17.01 | 17.12 | 17.17 | 17.13 | 17.18 | 0.40 |
| MBP | 87.56 | 87.72 | 87.88 | 87.13 | 87.81 | 0.34 |
| DBP | 69.98 | 72.25 | 69.42 | 70.55 | 69.38 | 1.68 |
| SBP | 128.74 | 127.70 | 126.42 | 129.06 | 124.57 | 1.45 |
| Heart rate | 75.49 | 77.93 | 74.50 | 78.34 | 77.02 | 2.12 |
| Urine output | 2008.20 | 2022.54 | 2023.50 | 2026.35 | 2028.76 | 0.40 |
| WBC | 11.54 | 11.48 | 11.63 | 11.65 | 11.55 | 0.60 |
| RBC | 3.70 | 3.77 | 3.70 | 3.74 | 3.70 | 0.81 |
| Hemoglobin | 10.96 | 11.22 | 10.96 | 11.12 | 10.98 | 1.06 |
| Hematocrit | 33.32 | 33.95 | 33.24 | 33.57 | 33.17 | 0.94 |
| Platelet | 234.14 | 233.72 | 233.52 | 234.62 | 234.16 | 0.18 |
| BUN | 19.07 | 18.97 | 19.11 | 19.16 | 18.99 | 0.41 |
| Creatinine | 1.06 | 1.05 | 1.06 | 1.04 | 1.06 | 0.79 |
| Glucose | 134.69 | 134.14 | 134.37 | 133.79 | 134.04 | 0.25 |
| Sodium | 138.91 | 139.02 | 138.97 | 138.86 | 138.92 | 0.04 |
| Potassium | 4.15 | 4.13 | 4.16 | 4.14 | 4.16 | 0.33 |
| Calcium | 8.21 | 8.22 | 8.22 | 8.22 | 8.22 | 0.02 |
| Anion gap | 13.54 | 13.61 | 13.56 | 13.66 | 13.58 | 0.33 |
| Chloride | 104.07 | 104.33 | 104.15 | 104.35 | 104.25 | 0.11 |
| PT | 15.41 | 15.46 | 15.35 | 15.36 | 15.34 | 0.34 |
| PTT | 39.14 | 39.16 | 39.34 | 39.10 | 38.94 | 0.37 |
| INR | 1.39 | 1.41 | 1.40 | 1.39 | 1.39 | 0.61 |
| GCS | 14.85 | 14.82 | 14.85 | 14.83 | 14.82 | 0.09 |
| SOFA | 3.17 | 3.21 | 3.22 | 3.27 | 3.24 | 1.13 |

CV was calculated to evaluate the stability of the imputed values across these seeds, with lower values indicating higher robustness. Abbreviations: BUN, blood urea nitrogen; CV, coefficient of variation; DBP, diastolic blood pressure; GCS, Glasgow Coma Scale; INR, international normalized ratio; MBP, mean blood pressure; PT, prothrombin time; PTT, partial thromboplastin time; RBC, red blood cell count; SBP, systolic blood pressure; SOFA, Sequential Organ Failure Assessment; WBC, white blood cell count.

**Supplementary Figure 1** ROC curves of the optimal model evaluated on five MICE-imputed datasets. Abbreviations: AUC, area under the receiver operating characteristic curve; MICE, multiple imputation by chained equations; ROC, receiver operating characteristic.

**Supplementary Figure 2** Correlation heatmap of categorical predictors in the development cohort. Associations were quantified using Cramér’s V (range 0–1), with larger values indicating stronger relationships. Cell values denote the corresponding Cramér’s V statistics, and color intensity reflects the magnitude of association. Abbreviations: AKI, acute kidney injury; CRRT, continuous renal replacement therapy; ICS, inhaled corticosteroids; NMBA, neuromuscular blocking agent; NSAIDs, non-steroidal anti-inflammatory drugs.

**Supplementary Figure 3** Correlation heatmap of continuous predictors in the development cohort. Correlations were calculated using Spearman’s rank correlation coefficient (ρ) to account for non-normal distributions and potential monotonic relationships. Each cell reports the corresponding ρ value, with the color scale ranging from −1 (strong negative correlation) to +1 (strong positive correlation). Abbreviations: APSIII, Acute Physiology Score III; BUN, blood urea nitrogen; DBP, diastolic blood pressure; GCS, Glasgow Coma Scale; INR, international normalized ratio; LODS, Logistic Organ Dysfunction System; MBP, mean blood pressure; OASIS, Oxford Acute Severity of Illness Score; PTT, partial thromboplastin time; PT, prothrombin time; RBC, red blood cell; SAPSII, Simplified Acute Physiology Score II; SBP, systolic blood pressure; SIRS, Systemic Inflammatory Response Syndrome; SOFA, Sequential Organ Failure Assessment; WBC, white blood cell.

**Supplementary Figure 4** Stability of feature selection by Boruta and LASSO. (A) Confirmed frequency of each variable across five independent Boruta runs with different random seeds (1, 42, 100, 2023, and 9999). The red dashed line indicates the 80% threshold for robust selection. (B) Bootstrap selection probability of each variable from 100 LASSO iterations with 10-fold cross-validation at λ.1se. The red dashed line indicates the 50% threshold for stable selection. Variables exceeding both thresholds were intersected to determine the final predictor set.

**Supplementary Table 4** Hyperparameter tuning strategies and optimal settings for candidate machine-learning models.

| Models | Tuning method and metric | Optimal hyperparameters |
| --- | --- | --- |
| LR | Maximum Likelihood Estimation. | Not Applicable (Default); AIC = 4371. |
| SVM | 10-fold CV (caret); Grid Search. | sigma = 0.1, C = 1. |
| XGBoost | Grid search using an inner validation subset split from the training set. | max_depth = 3, eta = 0.05, nrounds = 150. |
| LightGBM | Grid Search with 10-fold CV. | num_leaves = 15, max_depth = -1, learning_rate = 0.1, n_estimators = 50. |
| NNet | Parallel Grid search using an inner validation subset split from the training set. | hidden =2. |
| RF | 10-fold CV (caret); Grid for mtry. | mtry = 2, ntree = 900. |
| KNN | 10-fold CV (caret). | kmax = 80, kernel = "triangular". |
| DT | 10-fold CV for cp. | cp = 0.0031, maxdepth = 6. |

Hyperparameter tunings are performed in the training set. Abbreviations: AIC, Akaike information criterion; CV, cross-validation; DT, Decision Tree; KNN, k-Nearest Neighbors; LightGBM, Light Gradient Boosting Machine; LR, Logistic Regression; NNet, Neural Network; RF, Random Forest; SVM, Support Vector Machine; XGBoost, Extreme Gradient Boosting.

**Supplementary Table 5** Baseline characteristics of the training cohort after random undersampling.

| Variables | Non-MDR (n=1893) | MDR (n=1893) | p-value |
| --- | --- | --- | --- |
| Gender, male, n(%) | 1101 (58.2) | 1005 (53.1) | 0.002 |
| Temperature, ℃ | 36.66 (0.79) | 36.80 (0.96) | <0.001 |
| Heart rate, bpm | 87.69 (19.22) | 93.54 (21.74) | <0.001 |
| Hemoglobin, g/dl | 10.96 (2.24) | 10.40 (2.24) | <0.001 |
| Platelet, k/ul | 205.84 (105.06) | 213.27 (125.60) | 0.048 |
| BUN, mg/dl | 22.81 (19.68) | 32.05 (26.21) | <0.001 |
| Liver disease, n(%) | 223 (11.8) | 399 (21.1) | <0.001 |
| Diabetes, n(%) | 551 (29.1) | 654 (34.5) | <0.001 |
| AKI stage (%) |  |  | <0.001 |
| 0 | 585 (30.9) | 295 (15.6) |  |
| 1 | 370 (19.5) | 244 (12.9) |  |
| 2 | 633 (33.4) | 655 (34.6) |  |
| 3 | 305 (16.1) | 699 (36.9) |  |
| SIRS | 2.58 (0.95) | 2.86 (0.91) | <0.001 |
| APSIII | 41.60 (19.53) | 54.27 (22.59) | <0.001 |
| NMBA, n(%) | 120 (6.3) | 357 (18.9) | <0.001 |
| Statins, n(%) | 663 (35.0) | 486 (25.7) | <0.001 |
| Systemic glucocorticoids, n(%) | 392 (20.7) | 574 (30.3) | <0.001 |
| CRRT, n(%) | 70 (3.7) | 254 (13.4) | <0.001 |
| Enteral nutrition, n(%) | 252 (13.3) | 789 (41.7) | <0.001 |
| Parenteral nutrition, n(%) | 12 (0.6) | 108 (5.7) | <0.001 |
| Peripheral Lines, n(%) | 1652 (87.3) | 1577 (83.3) | 0.001 |
| Opioids, n(%) | 860 (45.4) | 1283 (67.8) | <0.001 |

**Abbreviations:** MDR, multidrug-resistant; AKI, acute kidney injury; BUN, blood urea nitrogen; SIRS, systemic inflammatory response syndrome; APSIII, Acute Physiology Score III; NMBA, neuromuscular blocking agent; CRRT, continuous renal replacement therapy. The undersampled training cohort was generated after feature selection by randomly selecting patients from the majority class to match the number of patients in the minority class. Only the final selected predictors used for model development are shown in this table.

**Supplementary Table 6** Baseline characteristics of MIMIC-III cohort stratified by MDR status.

| Variables | Non-MDR (n=34190) | MDR (n=4393) | p-value |
| --- | --- | --- | --- |
| Age, years | 62.65 (16.44) | 64.18 (15.42) | <0.001 |
| Gender, male, n(%) | 19775 (57.8) | 2445 (55.7) | 0.006 |
| Race, n(%) |  |  | 0.688 |
| White | 24303 (71.1) | 3097 (70.5) |  |
| Black | 3263 (9.5) | 435 (9.9) |  |
| Yellow | 798 (2.3) | 112 (2.5) |  |
| Others | 5826 (17.0) | 749 (17.0) |  |
| Marital status, n(%) |  |  | <0.001 |
| Single | 8789 (25.7) | 1178 (26.8) |  |
| Married | 17034 (49.8) | 2001 (45.5) |  |
| Divorced | 2136 (6.2) | 354 (8.1) |  |
| Widowed | 4117 (12.0) | 571 (13.0) |  |
| Unknown | 2114 (6.2) | 289 (6.6) |  |
| Heart rate, bpm | 103.55 (20.20) | 108.73 (22.29) | <0.001 |
| SBP, mmHg | 92.28 (17.51) | 88.70 (17.70) | <0.001 |
| DBP, mmHg | 44.22 (11.47) | 41.39 (11.75) | <0.001 |
| MBP, mmHg | 58.88 (13.16) | 55.87 (14.26) | <0.001 |
| Respiratory rate, bpm | 27.17 (6.45) | 28.33 (6.96) | <0.001 |
| Temperature, ℃ | 37.52 (0.75) | 37.67 (0.89) | <0.001 |
| Weight, kg | 81.19 (22.60) | 83.03 (27.14) | <0.001 |
| Urine output, ml | 2091.59 (3336.10) | 1740.94 (1256.28) | <0.001 |
| WBC, K/uL | 11.92 (9.48) | 13.09 (9.17) | <0.001 |
| RBC, m/uL | 3.59 (0.71) | 3.44 (0.67) | <0.001 |
| Hematocrit, % | 31.88 (5.88) | 30.80 (5.64) | <0.001 |
| Hemoglobin, mg/dl | 10.81 (2.01) | 10.29 (1.90) | <0.001 |
| Platelet, K/uL | 216.37 (108.83) | 230.63 (134.11) | <0.001 |
| BUN, mg/dl | 24.37 (20.42) | 32.79 (25.36) | <0.001 |
| Creatinine, mg/dl | 1.39 (1.59) | 1.67 (1.67) | <0.001 |
| Potassium, mEq/L | 4.18 (0.70) | 4.20 (0.81) | 0.032 |
| Sodium, mEq/L | 138.70 (4.54) | 138.99 (5.55) | <0.001 |
| Calcium, mEq/L | 8.27 (0.81) | 8.16 (0.97) | <0.001 |
| Chloride, mEq/L | 105.56 (6.10) | 105.12 (7.00) | <0.001 |
| Anion gap, mEq/L | 13.86 (3.80) | 14.67 (4.11) | <0.001 |
| Glucose, mg/dl | 141.87 (71.10) | 148.17 (78.92) | <0.001 |
| INR | 1.55 (1.09) | 1.71 (1.12) | <0.001 |
| PT, s | 16.10 (6.13) | 17.08 (7.20) | <0.001 |
| PTT, s | 39.50 (20.90) | 41.32 (22.25) | <0.001 |
| Congestive heart failure, n(%) | 9044 (26.5) | 1606 (36.6) | <0.001 |
| Chronic pulmonary disease, n(%) | 7411 (21.7) | 1178 (26.8) | <0.001 |
| Diabetes, n(%) | 9252 (27.1) | 1325 (30.2) | <0.001 |
| Renal disease, n(%) | 5129 (15.0) | 894 (20.4) | <0.001 |
| Liver disease, n(%) | 3434 (10.0) | 691 (15.7) | <0.001 |
| Malignant cancer, n(%) | 963 (2.8) | 138 (3.1) | 0.242 |
| AKI, n(%) | 22329 (65.3) | 3572 (81.3) | <0.001 |
| AKI stage, n(%) |  |  | <0.001 |
| 1 | 6164 (18.0) | 574 (13.1) |  |
| 2 | 10925 (32.0) | 1494 (34.0) |  |
| 3 | 5240 (15.3) | 1504 (34.2) |  |
| GCS | 13.78 (2.62) | 13.62 (2.72) | <0.001 |
| SOFA | 4.05 (2.93) | 5.44 (3.44) | <0.001 |
| SIRS | 2.75 (0.99) | 3.00 (0.94) | <0.001 |
| APSIII | 42.15 (19.06) | 52.60 (21.00) | <0.001 |
| LODS | 3.89 (2.58) | 5.16 (2.83) | <0.001 |
| OASIS | 31.11 (8.67) | 35.56 (8.82) | <0.001 |
| SAPSII | 33.90 (13.67) | 40.80 (13.56) | <0.001 |
| Vasoactive, n(%) | 9829 (28.7) | 1774 (40.4) | <0.001 |
| ICS, n(%) | 1694 (5.0) | 350 (8.0) | <0.001 |
| Systemic glucocorticoids, n(%) | 5671 (16.6) | 1284 (29.2) | <0.001 |
| Statins, n(%) | 9409 (27.5) | 821 (18.7) | <0.001 |
| NSAIDs, n(%) | 22795 (66.7) | 2944 (67.0) | 0.661 |
| Opioids, n(%) | 22028 (64.4) | 3170 (72.2) | <0.001 |
| Sedative, n(%) | 18961 (55.5) | 2905 (66.1) | <0.001 |
| NMBA, n(%) | 1511 (4.4) | 664 (15.1) | <0.001 |
| Biological agent, n(%) | 80 (0.2) | 28 (0.6) | <0.001 |
| Immunosuppressor, n(%) | 1054 (3.1) | 257 (5.9) | <0.001 |
| CRRT, n(%) | 543 (1.6) | 292 (6.6) | <0.001 |
| Ventilation, n(%) | 7792 (22.8) | 1937 (44.1) | <0.001 |
| Enteral nutrition, n(%) | 3331 (9.7) | 1834 (41.7) | <0.001 |
| Parenteral nutrition, n(%) | 1169 (3.4) | 845 (19.2) | <0.001 |
| Invasive Lines, n(%) | 9976 (29.2) | 1183 (26.9) | 0.002 |
| Peripheral Lines, n(%) | 14782 (43.2) | 1319 (30.0) | <0.001 |
| Los hospital, days | 9.21 (7.94) | 23.08 (20.50) | <0.001 |
| Hospital mortality, n(%) | 3175 (9.3) | 906 (20.6) | <0.001 |
| Los ICU, days | 3.82 (4.39) | 11.47 (12.65) | <0.001 |
| ICU mortality, n(%) | 2319 (6.8) | 603 (13.7) | <0.001 |
| 30-day survival time, days | 27.47 (7.38) | 26.54 (7.73) | <0.001 |
| 30-day mortality, n(%) | 4066 (11.9) | 906 (20.6) | <0.001 |
| 90-day survival time, days | 78.81 (27.09) | 69.86 (31.82) | <0.001 |
| 90-day mortality, n(%) | 5590 (16.3) | 1436 (32.7) | <0.001 |
| 180-day survival time, days | 152.44 (59.51) | 126.92 (71.45) | <0.001 |
| 180-day mortality, n(%) | 6762 (19.8) | 1743 (39.7) | <0.001 |

Abbreviations: AKI, acute kidney injury; APSIII, Acute Physiology Score III; BUN, blood urea nitrogen; CRRT, continuous renal replacement therapy; DBP, diastolic blood pressure; GCS, Glasgow Coma Scale; ICS, inhaled corticosteroids; ICU, intensive care unit; INR, international normalized ratio; LOS, length of stay; LODS, Logistic Organ Dysfunction System; MBP, mean blood pressure; MDR, multidrug-resistant; NMBA, neuromuscular blocking agents; NSAIDs, non-steroidal anti-inflammatory drugs; OASIS, Oxford Acute Severity of Illness Score; PT, prothrombin time; PTT, partial thromboplastin time; RBC, red blood cell count; SAPSII, Simplified Acute Physiology Score II; SBP, systolic blood pressure; SIRS, Systemic Inflammatory Response Syndrome; SOFA, Sequential Organ Failure Assessment; WBC, white blood cell count.

**Supplementary Table 7** Performance comparison of eight prediction models for multidrug-resistant (MDR) infections in the undersampling validation set.

| Model | AUC | AUPRC | Sensitivity | Specificity | F1 | Balanced accuracy | MCC | Kappa | Log Loss | ECE | HL p-value | Brier |
| --- | --- | --- | --- | --- | --- | --- | --- | --- | --- | --- | --- | --- |
| LR | 0.780 (0.758-0.802) | 0.754 (0.727-0.783) | 0.686 (0.652-0.716) | 0.736 (0.706-0.765) | 0.703 (0.677-0.728) | 0.711 (0.689-0.732) | 0.422 (0.379-0.465) | 0.422 (0.378-0.464) | 0.573 (0.549-0.596) | 0.0516 (0.0382-0.0739) | 0.000 | 0.193 |
| KNN | 0.777 (0.755-0.799) | 0.757 (0.728-0.787) | 0.664 (0.631-0.696) | 0.744 (0.713-0.774) | 0.692 (0.665-0.717) | 0.704 (0.684-0.726) | 0.410 (0.368-0.457) | 0.408 (0.366-0.456) | 0.570 (0.547-0.591) | 0.0343 (0.0271-0.0584) | 0.047 | 0.194 |
| DT | 0.748 (0.725-0.771) | 0.722 (0.694-0.750) | 0.655 (0.622-0.686) | 0.732 (0.702-0.763) | 0.682 (0.655-0.706) | 0.694 (0.672-0.715) | 0.389 (0.344-0.431) | 0.388 (0.343-0.430) | 0.590 (0.568-0.612) | 0.0200 (0.0144-0.0458) | 0.128 | 0.201 |
| RF | 0.785 (0.763-0.807) | 0.753 (0.722-0.781) | 0.703 (0.671-0.732) | 0.720 (0.690-0.752) | 0.709 (0.684-0.734) | 0.711 (0.690-0.733) | 0.423 (0.377-0.470) | 0.423 (0.377-0.469) | 0.558 (0.534-0.581) | 0.0234 (0.0187-0.0499) | 0.579 | 0.189 |
| XGBoost | 0.785 (0.763-0.807) | 0.760 (0.734-0.789) | 0.714 (0.681-0.744) | 0.696 (0.666-0.728) | 0.707 (0.680-0.733) | 0.705 (0.682-0.728) | 0.410 (0.366-0.456) | 0.410 (0.366-0.456) | 0.561 (0.538-0.583) | 0.0385 (0.0303-0.0632) | 0.020 | 0.189 |
| LightGBM | 0.778 (0.756-0.801) | 0.761 (0.735-0.785) | 0.715 (0.684-0.747) | 0.694 (0.663-0.725) | 0.708 (0.683-0.733) | 0.705 (0.683-0.727) | 0.410 (0.367-0.456) | 0.410 (0.367-0.455) | 0.564 (0.541-0.587) | 0.0259 (0.0214-0.0544) | 0.161 | 0.191 |
| SVM | 0.763 (0.740-0.786) | 0.720 (0.689-0.748) | 0.731 (0.698-0.761) | 0.681 (0.652-0.712) | 0.713 (0.687-0.737) | 0.706 (0.684-0.727) | 0.412 (0.369-0.461) | 0.412 (0.368-0.460) | 0.582 (0.559-0.604) | 0.0319 (0.0236-0.0577) | 0.032 | 0.198 |
| Nnet | 0.776 (0.754-0.799) | 0.745 (0.715-0.772) | 0.703 (0.672-0.734) | 0.719 (0.688-0.749) | 0.709 (0.683-0.733) | 0.711 (0.689-0.732) | 0.422 (0.381-0.468) | 0.422 (0.380-0.468) | 0.573 (0.549-0.597) | 0.0434 (0.0349-0.0687) | 0.001 | 0.193 |

The AUPRC was employed to assess model performance under class imbalance, while MCC and Kappa were provided as robust measures of classification agreement between predictions and actual outcomes. To evaluate model calibration, the HL p-value (Hosmer-Lemeshow test) was used, where a value > 0.05 indicates acceptable calibration; ECE, Log Loss, and Brier score were further utilized to quantify the probabilistic accuracy, with lower values representing superior alignment between predicted probabilities and observed frequencies. **Abbreviations:** AUC, area under the receiver operating characteristic curve; AUPRC, area under the precision-recall curve; Brier, Brier score; DT, Decision Tree; ECE, expected calibration error; HL p-value, Hosmer-Lemeshow p-value; Kappa, Cohen’s kappa coefficient; KNN, K-Nearest Neighbors; LightGBM, Light Gradient Boosting Machine; LR, Logistic Regression; MCC, Matthews correlation coefficient; Nnet, Neural Network; RF, Random Forest; SVM, Support Vector Machine; XGBoost, Extreme Gradient Boosting.

**Supplementary Figure 5** Discrimination and calibration of eight prediction models for multidrug-resistant (MDR) infections in the undersampling validation set. (A) Receiver operating characteristic (ROC) curves comparing the discriminative performance of Logistic Regression, K-Nearest Neighbors, Decision Tree, Random Forest, Extreme Gradient Boosting, Light Gradient Boosting Machine, Support Vector Machine, and Neural Network. (B) Calibration curves showing agreement between predicted probability and observed probability for each model. Abbreviations: AUC, area under the receiver operating characteristic curve; DT, decision tree; KNN, K-Nearest Neighbors; LightGBM, Light Gradient Boosting Machine; LR, Logistic Regression; MDR, multidrug-resistant; NNet, Neural Network; ROC, receiver operating characteristic; RF, Random Forest; SVM, Support Vector Machine; XGBoost, Extreme Gradient Boosting.

**Supplementary Figure 6** Decision-analytic evaluation of model utility in the undersampling validation set. (A) Decision curve analysis (DCA) comparing the standardized net benefit of eight prediction models across a range of high-risk threshold probabilities. The gray reference lines indicate default strategies (treat-none and treat-all). (B) Clinical impact curve (CIC) for the Random Forest model showing the number of patients classified as high risk (solid line) and the corresponding number of true MDR events among those labeled high risk (dashed line) at each threshold. Abbreviations: CIC, clinical impact curve; DCA, decision curve analysis; MDR, multidrug-resistant; XGBoost, extreme gradient boosting.

**Supplementary Table 8** Distribution of MDR status across microbiological subgroups in the MIMIC-IV cohort.

| Subgroup | MDR | Count | Ratio |
| --- | --- | --- | --- |
| CR (N=45,934) | no | 45,790 | 0.9969 |
| CR (N=45,934) | yes | 144 | 0.0031 |
| *Klebsiella* spp*.* (N=45,972) | no | 45,790 | 0.9960 |
| *Klebsiella* spp*.* (N=45,972) | yes | 182 | 0.0040 |
| *Pseudomonas* spp*.* (N=45,940) | no | 45,790 | 0.9967 |
| *Pseudomonas* spp*.* (N=45,940) | yes | 150 | 0.0033 |

Abbreviations: CR, carbapenem-resistant organisms; MDR, multidrug-resistant; N, total number of patients in each subgroup; Ratio, proportion of MDR or non-MDR patients within each subgroup.

**Supplementary Figure 7** Pathogen-specific MDR analyses of the Random Forest model across clinically relevant MDR pathogen categories in the total study cohort. ROC curves showing the discriminative ability of the RF model for three clinically relevant MDR pathogen categories, each compared against the original non-MDR group. (A) Carbapenem-resistant organism infections (AUC = 0.858, 95% CI 0.833–0.882); (B) *Klebsiella* spp. infections (AUC = 0.822, 95% CI 0.797–0.846); (C) *Pseudomonas* spp. infections (AUC = 0.835, 95% CI 0.809–0.861). Abbreviations: AUC, area under the receiver operating characteristic curve; CI, confidence interval; MDR, multidrug-resistant; RF, Random Forest; ROC, receiver operating characteristic.

**Supplementary Table 9** Distribution of MDR status across microbiological subgroups in the internal validation cohort.

| Subgroup | MDR | Count | Ratio |
| --- | --- | --- | --- |
| CR (N=13,766) | no | 13,732 | 0.9975 |
| CR (N=13,766) | yes | 34 | 0.0025 |
| Klebsiella spp. (N=13,790) | no | 13,732 | 0.9958 |
| Klebsiella spp. (N=13,790) | yes | 58 | 0.0042 |
| Pseudomonas spp. (N=13,774) | no | 13,732 | 0.997 |
| Pseudomonas spp. (N=13,774) | yes | 42 | 0.003 |

Abbreviations: CR, carbapenem-resistant organisms; MDR, multidrug-resistant; N, total number of patients in each subgroup; Ratio, proportion of MDR or non-MDR patients within each subgroup.

**Supplementary Figure 8** Pathogen-specific MDR analyses of the Random Forest model in the internal validation set. ROC curves showing the discriminative ability of the RF model for three clinically relevant MDR pathogen categories, each compared against the original non-MDR group. (A) Carbapenem-resistant organism infections (AUC = 0.849, 95% CI 0.794–0.904); (B) *Klebsiella* spp. infections (AUC = 0.823, 95% CI 0.779–0.867); (C) *Pseudomonas* spp. infections (AUC = 0.870, 95% CI 0.825–0.916). Abbreviations: AUC, area under the receiver operating characteristic curve; CI, confidence interval; MDR, multidrug-resistant; RF, Random Forest; ROC, receiver operating characteristic.

**Supplementary Figure 9** Discriminative performance of the Random Forest model at the 48-hour and 72-hour time windows after ICU admission in the internal validation set. ROC curves showing the discriminative ability of the RF model for MDR, each compared against the original non-MDR group. (A) 48-hour window, AUC = 0.741 (95% CI 0.713–0.768); (B) 72-hour window, AUC = 0.722 (95% CI 0.692–0.751). Abbreviations: AUC, area under the receiver operating characteristic curve; CI, confidence interval; MDR, multidrug-resistant; RF, Random Forest; ROC, receiver operating characteristic.

**Supplementary Figure 10** SHAP dependence plots for continuous predictors in the best-performing model. SHAP values were calculated for the uncalibrated model output. Each panel shows the univariate SHAP dependence relationship between a continuous predictor and its SHAP value. Each dot represents one patient. The x-axis is the observed value of the predictor, and the y-axis is the corresponding SHAP value, indicating how that value shifts the model output toward higher (positive SHAP) or lower (negative SHAP) predicted MDR risk. Plots are shown for APSIII, BUN, hemoglobin, platelet count, heart rate, temperature and SIRS. **Abbreviations:** APSIII, Acute Physiology Score III; BUN, blood urea nitrogen; SHAP, SHapley Additive exPlanations; SIRS, systemic inflammatory response syndrome.
